# Elucidating the impact of the pneumococcal conjugate vaccine programme on pneumonia, sepsis and otitis media hospital admissions in England using a composite control

**DOI:** 10.1186/s12916-018-1004-z

**Published:** 2018-02-08

**Authors:** Dominic Thorrington, Nick Andrews, Julia Stowe, Elizabeth Miller, Albert Jan van Hoek

**Affiliations:** 1 Public Health England, London, UK; 2 London School of Hygiene and Tropical Medicine, London, UK

**Keywords:** Pneumococcal conjugate vaccine, Composite controls, Pneumonia, Sepsis, Otitis media, Invasive pneumococcal disease, Impact assessment, Herd immunity, Indirect protection, Hospital Episode Statistics, Post vaccination, Vaccine impact, Non-invasive disease

## Abstract

**Background:**

The seven-valent pneumococcal conjugate vaccine (PCV) was introduced in England in September 2006, changing to the 13-valent vaccine in April 2010. PCV impact on invasive pneumococcal disease (IPD) has been extensively reported, but less described is its impact on the burden of pneumonia, sepsis and otitis media in the hospital.

**Methods:**

Using details on all admissions to hospitals in England, we compared the incidence of pneumococcal-specific and syndromic disease endpoints in a 24-month pre-PCV period beginning April 2004 to the 24-month period ending March 2015 to derive incidence rate ratios (IRRs). To adjust for possible secular trends in admission practice, IRRs were compared to the IRRs for five control conditions over the same period and the relative change assessed using the geometric mean of the five control IRRs as a composite, and individually for each control condition to give the min-max range. Relative changes were also compared with IRRs for IPD from the national laboratory database. The effect of stratifying cases into those with and without clinical risk factors for pneumococcal infection was explored.

**Results:**

Relative reductions in pneumococcal pneumonia were seen in all age groups and in those with and without risk factors; in children under 15 years old reductions were similar in magnitude to reductions in IPD. For pneumonia of unspecified cause, relative reductions were seen in those under 15 years old (maximum reduction in children under 2 years of 34%, min-max: 11–49%) with a relative increase in 65+ year olds most marked in those with underlying risk conditions (41%, min-max: 0–82%). Reductions in pneumococcal sepsis were seen in all age groups, with the largest reduction in children younger than 2 years (67%, min-max 56–75%). Reductions in empyema and lung abscess were also seen in under 15 year olds. Results for other disease endpoints were varied. For disease endpoints showing an increase in raw IRR, the increase was generally reduced when expressed as a relative change.

**Conclusions:**

Use of a composite control and stratification by risk group status can help elucidate the impact of PCV on non-IPD disease endpoints and in vulnerable population groups. We estimate a substantial reduction in the hospitalised burden of pneumococcal pneumonia in all age groups and pneumonia of unspecified cause, empyema and lung abscess in children under 15 years of age since PCV introduction. The increase in unspecified pneumonia in high-risk 65+ year olds may in part reflect their greater susceptibility to develop pneumonia from less pathogenic serotypes that are replacing vaccine types in the nasopharynx.

**Electronic supplementary material:**

The online version of this article (doi:10.1186/s12916-018-1004-z) contains supplementary material, which is available to authorized users.

## Background

*Streptococcus pneumoniae* is a common gram-positive bacterial pathogen with more than 90 identified serotypes that may reside in the nasopharynx of healthy individuals. It can cause diseases such as otitis media and pneumonia if it spreads to adjacent organs. If the bacteria enter the bloodstream, they cause invasive pneumococcal disease (IPD) manifesting as bacteraemic pneumonia, meningitis or other clinical presentations. Pneumococci are transmitted by direct contact with respiratory secretions from both healthy carriers and patients [1].

The burden of disease is not evenly distributed, with those individuals with certain chronic conditions (immunosupression, cochlear implants, asthma, diabetes, alcoholism, chronic diseases of the lungs, heart, liver, kidneys) at a much greater risk of infection [2].

Many developed countries have implemented a pneumococcal vaccination programme targeting different sectors of the population to protect against a number of the most prevalent serotypes. In England, the 23-valent polysaccharide vaccine (PPV23) was first recommended for those in clinical risk groups in 1992, then extended to elderly cohorts in a step-wise manner, eventually being offered to all individuals aged 65+ years in 2003. Uptake for 2014–2015 was 69.8% of eligible elderly individuals [3]. The seven-valent conjugate vaccine (PCV7) was first offered to all infants in September 2006 as a 2 + 1 schedule (at 2, 4 and 12 months), with a catch-up campaign for children up to 2 years old. PCV7 was replaced in April 2010 by the 13-valent conjugate vaccine (PCV13) on the same schedule but without a catch-up campaign.

Analyses in England have demonstrated the effectiveness [4] and population impact of the sequential PCV7 and PCV13 vaccination programme in infants through the reduction of vaccine-type carriage [5, 6] and IPD caused by the serotypes targeted by the vaccines [7, 8]. The impact of this programme has also been demonstrated in terms of indirect protection against IPD provided to unvaccinated cohorts, though serotype replacement with non-vaccine types has reduced the overall impact on IPD [7, 8].

We present an analysis of the estimated direct and indirect impact of the PCV programme on hospital-diagnosed pneumonia, sepsis and otitis media on all ages using Hospital Episodes Statistics (HES) data from 2004–2005 to 2014–2015. We compare the disease trends for these outcomes to trends for five control conditions unlikely to be affected by the PCV vaccination to infer both direct and indirect impact of the PCV programme.

## Methods

### Identification of cases

Public Health England has access to the HES database. This electronic database contains data on all episodes of hospital care in England. An episode is defined as a continuous period of care administered within a particular consultant specialty at a single hospital provider. HES uses the International Statistical Classification of Diseases and Related Health Problems – 10^th^ Revision (ICD-10) classification to record diagnoses, of which there are up to 20 fields available to clinicians, and can therefore provide information on the burden of hospitalised disease due to different pathogens or syndromes in England. We extracted data from HES for the period April 2004 to March 2015 for all admissions with a pneumonia, sepsis or otitis media ICD-10 code in any diagnosis field for individuals in seven age groups: less than 2 years old, 2–4 years, 5–14, 15–24, 25–44, 45–64 and 65+. The main analysis was restricted to admissions with an ICD-10 code of interest in the first diagnosis field, as this field indicates the primary cause of the admission. Outpatient appointments and emergency room consultations were not available. Admissions were allocated to a HES data year (April to March) using the date of discharge.

Our analysis of the impact of PCV on pneumonia focused on pneumococcal pneumonia (J13) and pneumonia of unspecified causative organism (J18). J13 is a bacterial pneumonia for which the cause is identified as the pneumococcus. J18 is the most common of the pneumonia diagnoses and has a mixed but unknown aetiology. Respiratory infections mentioning pneumonia associated with influenza or other viruses (J09-12) or other specific bacterial causes of pneumonia (J14-J17) were excluded We also considered the impact on other respiratory conditions in which the pneumococcus can have a causative role, namely empyema (J869) and abscess of lung with pneumonia (J851). We identified sepsis diagnoses using the list of ICD-10 codes reported in a cluster-randomised trial assessing the impact of PCV10 on suspected invasive pneumococcal disease in Finland [9], with pneumococcal-specific sepsis identified using the diagnosis codes of A403, B953, G001 and M001 and non-specific sepsis identified using the diagnosis codes A409, A419, A491, A499, B955, G009, M009 and I301. We identified otitis media using the H65, H66 and H67 ICD-10 codes, and we also identified a subgroup of otitis media cases with procedure codes relating to the insertion, removal or maintenance of ventilation tubes through the tympanic membrane using the Office of Population Censuses and Surveys, Classification of Surgical Operations and Procedures-4th revision (OPCS-4) procedure codes D151, D202 and D203 to assess the impact of the PCV programme on recurrent acute otitis media. The ICD-10 codes used to identify admissions are listed in Additional file 1: Table S1. Repeat admissions within 90 days in the same individual and with the same ICD-10 code in the first field were considered to be the same disease episode. Incidence per 100,000 person-years was estimated using mid-year population estimates for England for 2004 to 2015 from the Office for National Statistics as the denominator [10].

### Identification of risk groups

Risk groups were identified using ICD-10 codes for comorbidities found to increase the risk of pneumococcal infection in any of the 20 diagnosis fields [2] (immunosupression, cochlear implants, asthma, diabetes, alcoholism, and chronic diseases of the lungs, heart, liver and kidneys; all ICD-10 codes are listed in Additional file 1: Table S2).

### Assessing the impact of the PCV programme

Using reports from diagnostic laboratories in England and Wales to the national surveillance centre at Public Health England, we compared trends in annual hospitalisations for each disease endpoint to the incidence of IPD diagnosed by culture of *S. pneumoniae* from a normally sterile site, or by antigen detection or polymerase chain reaction in cerebrospinal or pleural fluid. The IPD laboratory data were adjusted for the improvements in the efficiency of laboratory reporting of bacteraemias up to 2009–2010, as well as for reports with missing age information [7, 8]. Incidence per 100,000 person-years for laboratory-confirmed IPD was estimated using mid-year population estimates for England and Wales for 2004 to 2015 from the same source as above [10]. We estimated the change in incidence for laboratory-confirmed IPD and each hospitalised disease endpoint by calculating incidence rate ratios (IRRs) using two defined time periods: the pre-PCV era (1 April 2004–31 March 2006) to match the same calendar period in the post-PCV era (1 April 2013–31 March 2015), which was the most recent 2-year time period in the dataset at the time of extraction.

To assess the robustness of our estimates of the IRRs, we performed a sensitivity analysis by calculating the IRRs for each disease endpoint but expanding the number of ICD-10 fields to the first three diagnoses and then using all diagnoses available, while selection of controls remained unchanged.

To account for biases arising from potential secular trends in admission practice over the study period, the IRRs of each disease endpoint were compared to the IRRs of five control conditions that should not be affected by changes in the introduction of the PCV programme [11]. These were urinary tract infections, infections of the skin and subcutaneous tissue, disorders of the thyroid gland, diseases of the blood, and fractures. These conditions were selected a priori with the criteria that they were not caused by the pneumococcus; not the focus of other public health interventions; and with a large case burden with a similar age distribution of cases to the pneumococcal disease outcomes. We took admissions with the appropriate ICD-10 code in the first diagnosis code of a patient's episode (ICD-10s listed in Additional file 1: Table S3). We excluded admissions for the control conditions if one of the concurrent diagnoses was for one of the studied diseases listed in Additional file 1: Table S1. Readmissions within 90 days with the same ICD-10 code in the first field were considered to be the same disease episode.

We calculated the age-specific ratio of the IRRs (denoted rIRR) for each disease over each control condition. This ratio was used to help assess the impact of the PCV programme as follows:

- Equal to 1 implies no difference in activity between the disease endpoint and the control condition.
- Greater than 1 implies an increase in pneumococcal disease incidence post-PCV greater than that seen in the control, or a decrease less than that seen in the control.
- Less than 1 implies a decrease in pneumococcal disease incidence post-PCV greater than that seen in the control, or an increase less than that seen in the control.

In addition, the age-specific IRRs of the disease endpoints were compared to a composite control, calculated using the geometric mean of the age-specific IRRs of all five control conditions. The rIRR using the composite control is presented along with the minimum and maximum rIRRs calculated for each of the five control conditions separately as an indicator of uncertainty. We used the rIRR measure to estimate the change in the absolute number of hospitalisations for each disease endpoint attributable to the PCV programme since April 2007, the first complete HES data year after vaccine introduction, by assuming that the IRR of each disease endpoint would have mirrored that of the composite control in the absence of the PCV programme.

In assessing the impact of the PCV programme on pneumonia by risk group status, since the size of the denominator population by age group and year with and without risk factors was not accurately known, we used the raw numbers of admissions to calculate case ratios for the pneumonia outcomes and the control conditions and then derived the ratio of the two case ratios (termed adjusted case ratios).

We did not compare the IRR of laboratory-reported IPD to the control conditions from HES because the IPD data from laboratories are not subject to the same secular trends as the HES data and were already corrected for trends in ascertainment of laboratory-confirmed pneumococcal bacteraemias [7, 8].

## Results

### Case numbers

The burden of pneumonia with unspecified causative organism across all ages (1,683,478 cases) was much larger than that of pneumococcal pneumonia (30,459 cases, Table 1). For sepsis cases, 97% (297,052/304,714) were attributed to non-specific sepsis diagnoses, with pneumococcal sepsis causing a small percentage (3%, 7976/304,714). More than 79% of all sepsis diagnoses were sepsis with unspecified organism (ICD-10: A419). Of the 408,999 otitis media admissions over the study period, 74% (108,598/408,999) were for nonsuppurative otitis media, with 315,908 of all otitis media cases with tympanostomies (77% of the total). A full breakdown by age of the number cases for each ICD-10 diagnosis is available in Additional file 1: Table S4.

**Table 1:** The number of cases of pneumonia, sepsis and otitis media in England and the percentage of those cases for individuals in clinical risk groups, from April 2004 through March 2015

ICD-10	Total number of cases (% cases in risk groups)
Pneumonia	
Pneumococcal pneumonia	30,459 (58%)
Pneumonia (unsp. organism)	1,683,478 (66%)
Total	1,713,937 (66%)
Remaining two respiratory conditions	
Empyema	23,434 (45%)
Lung abscess with pneumonia	2616 (58%)
Total	26,050 (47%)
Sepsis	
Any sepsis	304,714 (66%)
Any non-specific sepsis	297,052 (67%)
Any pneumococcal sepsis	7976 (43%)
Otitis media	
All otitis media	408,999 (5%)
Otitis media, tympanostomy	315,908 (4%)

Individuals in risk groups identified by presence of comorbidities made up 66% of all pneumonia cases, 47% of all other respiratory cases, 66% of all sepsis cases and 5% of all otitis media cases.

4% of all pneumococcal pneumonia, pneumonia of unspecified causative organism, lung abscess and empyema cases identified by the first ICD-10 code had concurrent sepsis codes and 0.1% had otitis media codes in the remaining ICD-10 codes. We found that 17% of all sepsis cases and 0.4% of all otitis media cases identified by the first ICD-10 code had concurrent pneumonia, empyema or lung abscess with pneumonia codes in the remaining ICD-10 codes. Information on the case numbers for those individuals with diagnoses for more than one pneumococcal disease endpoint is provided in Additional file 1: Table S5.

### Changes in the incidence of pneumococcal-specific and syndromic disease, compared with IPD

**Fig. 1:**
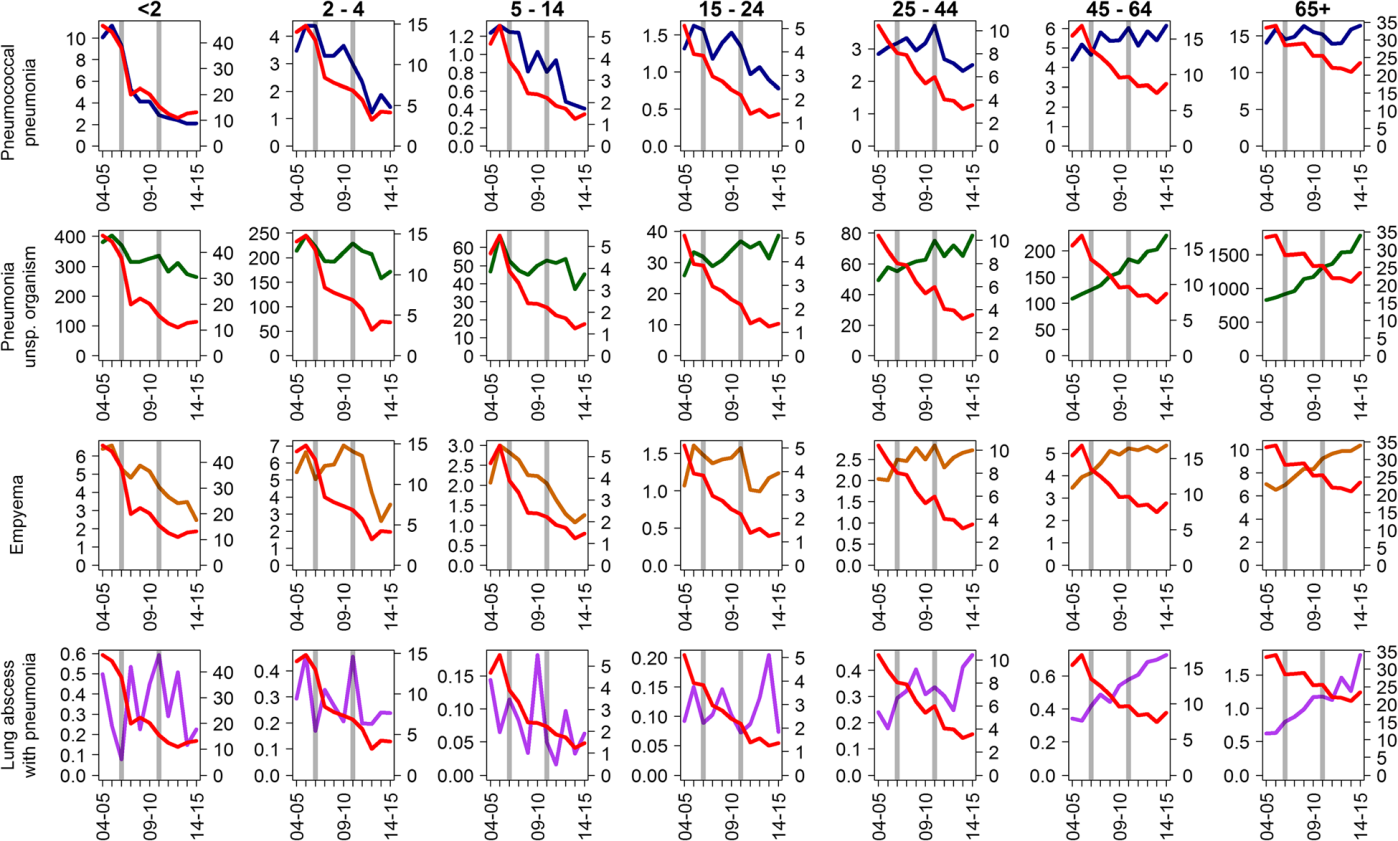
Comparison of the incidence per 100,000 of laboratory-confirmed IPD (plotted in *red*, *right-hand axes*) with the incidence per 100,000 of respiratory disease endpoints (*left-hand axes*)

Observed trends in incidence among the different respiratory disease endpoints did not consistently follow the reduction observed in the incidence of laboratory-reported IPD (Fig. 1). Disease trends for pneumococcal pneumonia were broadly similar to those reported for IPD for individuals up to 15–24 years of age, though not in older individuals, where IPD rates declined but pneumococcal pneumonia admissions did not. Reductions in admissions with pneumonia of unspecified causative organism were only evident in children up to 15 years of age; in the 65+ years age group the annual incidence of admission for J18 increased from 829 to 1787 per 100,000 person-years between 2004–05 and 2014–15 respectively. Reductions in admissions for empyema were also seen in children up to 15 years of age but not for admissions for lung abscess with pneumonia.

**Fig. 2:**
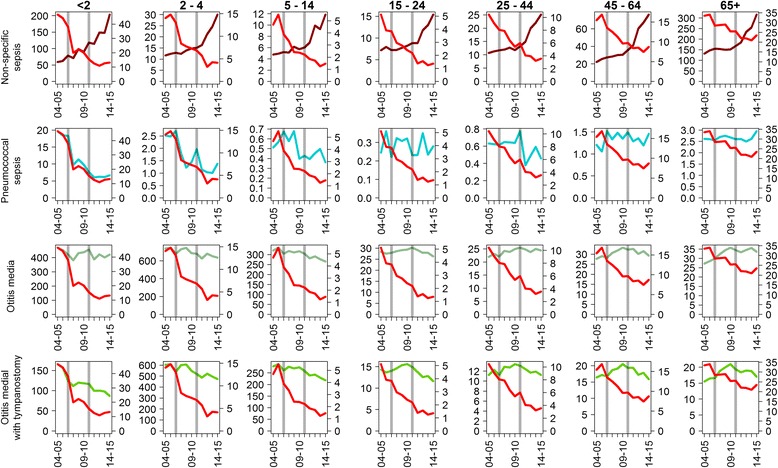
Comparison of the incidence per 100,000 of laboratory-confirmed IPD (plotted in *red*, *right-hand axes*) with the incidence per 100,000 of sepsis endpoints and otitis media endpoints (*left-hand axes*)

The incidence of pneumococcal sepsis declined from 20 to 7 per 100,000 person-years in the < 2 years age group between 2004–05 and 2014–15; declines were also seen in 2–14 year olds. In all age groups the incidence of non-specific sepsis increased over the same period (Fig. 2). The incidence of otitis media with tympanostomy showed a consistent decline in children under 15 years old, though a similar decline was not seen for all otitis media diagnoses, or in older age groups.

When added together the incidence of pneumococcal pneumonia and pneumococcal sepsis was lower than that of laboratory-confirmed IPD in all age groups (Figs. 1 and 2).

Table 2 shows both the IRRs for all studied disease endpoints compared with the IRR for IPD as well as the ratio of the IRRs for the disease endpoints compared with the composite control. The incidence of IPD for children younger than 2 years declined by 72% (95% confidence interval (CI) 68–75%) over the study period. For this age group the reduction in the incidence of pneumococcal pneumonia was 80% (95% CI 74–85%); reductions for other disease endpoints were recurrent acute otitis media requiring tympanostomy 47% (95% CI 44–50%), pneumonia of unspecified causative organism 31% (95% CI 29–33%) and empyema 54% (95% CI 40–65%). Reductions in pneumococcal pneumonia were also seen in age groups from 2–44 years, but increases were observed in individuals aged 45+ years. No reductions in incidence for this age group were seen for the other disease endpoints.

**Table 2:** The incidence rate ratios compare incidence between the pre-PCV era (1 April 2004–31 March 2006) and post-PCV era (1 April 2013–31 March 2015) for each disease endpoint. Also shown is ratio of incidence rate ratios (rIRRs) for each disease endpoint compared with the composite control, with the maximum and minimum denoting the range of the individual rIRRs using each of the five control conditions separately

Disease endpoint	IRR post pre-vaccination (95% CI)	rIRR disease endpoint, composite control [min, max]
<2 years		
IPD	0.28 (0.25–0.32)	
Pneumococcal pneumonia	0.20 (0.15–0.26)	0.19 [0.15, 0.25]
Pneumonia (unsp. organism)	0.69 (0.67–0.71)	0.66 [0.51, 0.89]
Empyema	0.46 (0.35–0.60)	0.44 [0.34, 0.59]
Lung abscess with pneumonia	0.50 (0.17–1.50)	0.49 [0.38, 0.65]
Non-specific sepsis	2.89 (2.72–3.06)	2.79 [2.15, 3.73]
Pneumococcal sepsis	0.34 (0.28–0.40)	0.33 [0.25, 0.44]
Otitis media	0.78 (0.76–0.81)	0.76 [0.58, 1.01]
Otitis media with tympanostomy	0.53 (0.50–0.56)	0.51 [0.39, 0.69]
2–4 years		
IPD	0.29 (0.24–0.34)	
Pneumococcal pneumonia	0.42 (0.31–0.56)	0.47 [0.36, 0.65]
Pneumonia (unsp. organism)	0.72 (0.70–0.74)	0.80 [0.62, 1.11]
Empyema	0.51 (0.41–0.63)	0.57 [0.44, 0.79]
Lung abscess with pneumonia	0.63 (0.28–1.44)	0.70 [0.54, 0.98]
Non-specific sepsis	2.28 (2.04–2.55)	2.54 [1.95, 3.52]
Pneumococcal sepsis	0.48 (0.34–0.68)	0.53 [0.41, 0.74]
Otitis media	0.82 (0.81–0.84)	0.92 [0.70, 1.27]
Otitis media with tympanostomy	0.79 (0.77–0.81)	0.88 [0.68, 1.22]
5–14 years		
IPD	0.27 (0.23–0.31)	
Pneumococcal pneumonia	0.34 (0.25–0.46)	0.31 [0.27, 0.41]
Pneumonia (unsp. organism)	0.73 (0.70–0.75)	0.67 [0.59, 0.87]
Empyema	0.46 (0.38–0.56)	0.43 [0.37, 0.56]
Lung abscess with pneumonia	0.45 (0.17–1.20)	0.42 [0.37, 0.55]
Non-specific sepsis	2.18 (1.98–2.40)	2.02 [1.76, 2.63]
Pneumococcal sepsis	0.79 (0.55–1.14)	0.73 [0.64, 0.96]
Otitis media	0.80 (0.78–0.81)	0.74 [0.64, 0.96]
Otitis media with tympanostomy	0.78 (0.77–0.80)	0.72 [0.63, 0.94]
15–24 years		
IPD	0.29 (0.25–0.34)	
Pneumococcal pneumonia	0.57 (0.45–0.72)	0.50 [0.40, 0.69]
Pneumonia (unsp. organism)	1.18 (1.13–1.23)	1.04 [0.84, 1.43]
Empyema	0.90 (0.72–1.11)	0.79 [0.64, 1.09]
Lung abscess with pneumonia	1.15 (0.59–2.23)	1.01 [0.82, 1.39]
Non-specific sepsis	1.87 (1.74–2.02)	1.65 [1.33, 2.27]
Pneumococcal sepsis	0.85 (0.54–1.33)	0.74 [0.60, 1.03]
Otitis media	0.86 (0.81–0.90)	0.75 [0.61, 1.04]
Otitis media with tympanostomy	0.85 (0.80–0.92)	0.75 [0.61, 1.04]
25– 44 years		
IPD	0.35 (0.32–0.37)	
Pneumococcal pneumonia	0.82 (0.74–0.90)	0.68 [0.52, 0.82]
Pneumonia (unsp. organism)	1.34 (1.31–1.36)	1.11 [0.86, 1.34]
Empyema	1.33 (1.19–1.48)	1.11 [0.85, 1.33]
Lung abscess with pneumonia	2.09 (1.54–2.83)	1.74 [1.34, 2.10]
Non-specific sepsis	2.14 (2.05–2.23)	1.78 [1.37, 2.15]
Pneumococcal sepsis	0.84 (0.68–1.04)	0.70 [0.54, 0.84]
Otitis media	0.98 (0.94–1.02)	0.82 [0.63, 0.98]
Otitis media with tympanostomy	0.97 (0.93–1.02)	0.81 [0.62, 0.98]
45–64 years		
IPD	0.50 (0.48–0.53)	
Pneumococcal pneumonia	1.21 (1.12–1.30)	0.91 [0.69, 1.03]
Pneumonia (unsp. organism)	1.90 (1.87–1.93)	1.44 [1.09, 1.63]
Empyema	1.41 (1.30–1.53)	1.07 [0.81, 1.21]
Lung abscess with pneumonia	2.13 (1.65–2.75)	1.61 [1.22, 1.83]
Non-specific sepsis	3.00 (2.92–3.09)	2.28 [1.73, 2.58]
Pneumococcal sepsis	1.17 (1.00–1.37)	0.89 [0.67, 1.01]
Otitis media	0.97 (0.94–1.01)	0.74 [0.56, 0.83]
Otitis media with tympanostomy	1.00 (0.95–1.04)	0.75 [0.57, 0.86]
65+ years		
IPD	0.66 (0.63–0.69)	
Pneumococcal pneumonia	1.08 (1.02–1.13)	0.87 [0.66, 1.03]
Pneumonia (unsp. organism)	1.97 (1.95–1.98)	1.58 [1.21, 1.88]
Empyema	1.49 (1.39–1.61)	1.20 [0.92, 1.43]
Lung abscess with pneumonia	2.44 (1.95–3.06)	1.96 [1.50, 2.33]
Non-specific sepsis	2.06 (2.03–2.09)	1.66 [1.26, 1.97]
Pneumococcal sepsis	1.07 (0.94–1.21)	0.86 [0.66, 1.02]
Otitis media	1.12 (1.07–1.17)	0.90 [0.69, 1.07]
Otitis media with tympanostomy	1.12 (1.07–1.19)	0.90 [0.69, 1.07]

No significant reduction was seen in the incidence of lung abscess with pneumonia for any age group. In contrast, admissions for empyema and pneumonia of unspecified causative organism reduced in all age groups under 15 years of age (Table 2).

### Using the composite control to estimate the ratio of IRRs

**Fig. 3:**
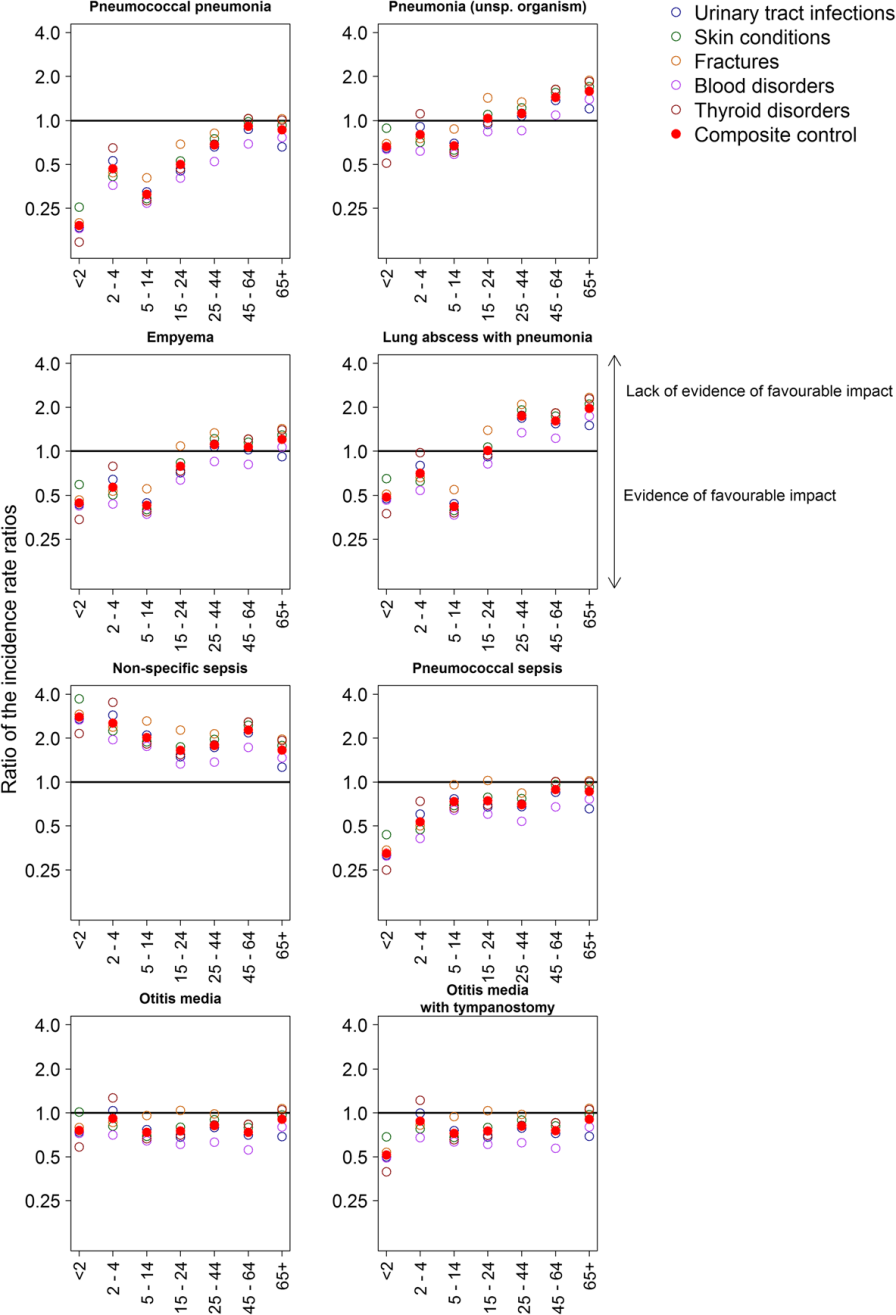
The ratio of incidence rate ratios, plotted on a log scale, for each age group for all eight disease endpoints against the control conditions. Points below the *horizontal line* indicate evidence of an impact of the PCV programme on the incidence of that disease endpoint

A reduction in the incidence of pneumococcal pneumonia compared to the composite control was observed in the < 2 years age group, in addition to reductions for all other endpoints except non-specific sepsis (Table 2 and Fig. 3). Compared to the composite control, reductions in the incidence of pneumococcal pneumonia and pneumococcal sepsis were seen in all age groups, though not when compared with each control condition individually (as evidenced by a value > 1 in the min, max range).

A favourable impact on pneumonia with unspecified causative organism was only evident in individuals aged less than 15 years, as the incidence of this endpoint increased more than the composite control for older age groups (Additional file 1: Figure S7). Reductions in the incidence of otitis media with tympanostomy were largest in children under 2 years of age (rIRR 0.51, min-max 0.39–0.69). Reductions were also seen at older ages, though not always in excess of those seen in all five of the control conditions.

In general, for those disease outcomes showing an increase in the raw IRR over the study period, the IRR when compared with the IRR of the composite control was lower.

### Sensitivity analysis

The magnitude of the rIRR varied with the number of ICD-10s used to identify each condition of interest. Using only the first ICD-10 code suggests that the incidence of pneumococcal pneumonia for the 65+ age group decreased by 13% (min-max -34–3%) relative to the composite control (Table 2), though expanding the number of diagnosis codes to the first three suggests a decrease in incidence of 23% (min-max 9–41%), and expanding the number of diagnosis codes for all suggests a decrease in incidence of 25% (min-max 11–43%) (Additional file 1: Table S6). In younger age groups, use of additional ICD-10 codes had little impact (25–44 years) or lessened the relative reduction in pneumococcal pneumonia (<25 years). For pneumonia of unspecified cause, increasing the number of ICD-10 codes generally increased the rIRR.

### Changes in pneumococcal pneumonia and pneumonia due to unspecified organism by risk group

To improve the understanding regarding the observed increase in unspecified pneumonia in older age groups, the data were split into those with and without ICD codes indicating a risk group. Table 3 shows both the raw case ratios and case ratios adjusted using the composite control for risk and non-risk groups for pneumococcal pneumonia and pneumonia of unspecified causative organism. For pneumococcal pneumonia, significant reductions were seen in adjusted case ratios in risk and non-risk groups of all ages (Additional file 1: Figures S8, S10). For unspecified pneumonia, increases in raw case ratios were found mainly among those in clinical risk groups (Additional file 1: Figures S9, S11), but as there was also an increase among the controls (Additional file 1: Table S8), there is a marked difference between the raw and adjusted case ratios. The adjusted case ratios in younger age groups were broadly similar between risk and non-risk groups up to the 5–14 years group, but there is a marked divergence in the older age groups.

**Table 3:** Age group- and risk group-specific ratios of case ratios (termed adjusted case ratios) for pneumococcal pneumonia and pneumonia of unspecified causative organism against the control conditions

	Pneumococcal pneumonia				Pneumonia of unspecified causative organism			
	Risk		Non-risk		Risk		Non-risk	
	Raw case ratio	Adjusted case ratio	Raw case ratio	Adjusted case ratio	Raw case ratio	Adjusted case ratio	Raw case ratio	Adjusted case ratio
<2 years	0.36	0.18 (0.14–0.25)	0.21	0.19 (0.15–0.25)	1.34	0.67 (0.50–0.92)	0.73	0.67 (0.51–0.87)
2–4	1.58	0.73 (0.58–0.97)	0.41	0.40 (0.32–0.59)	1.74	0.80 (0.64–1.06)	0.84	0.82 (0.67–1.22)
5–14	0.62	0.35 (0.25–0.46)	0.33	0.31 (0.28–0.39)	1.31	0.73 (0.53–0.96)	0.70	0.67 (0.60–0.84)
15–24	0.50	0.35 (0.27–0.50)	0.61	0.51 (0.38–0.72)	2.19	1.52 (1.20–2.19)	1.11	0.93 (0.69–1.30)
25–44	1.15	0.70 (0.59–0.79)	0.73	0.62 (0.45–0.74)	2.23	1.35 (1.15–1.53)	1.15	0.99 (0.72–1.18)
45–64	1.78	0.83 (0.66–0.96)	0.99	0.77 (0.56–0.85)	2.88	1.34 (1.07–1.56)	1.48	1.15 (0.83–1.27)
65+	1.61	0.77 (0.55–0.99)	0.61	0.61 (0.54–0.71)	2.96	1.41 (1.00–1.82)	1.13	1.13 (0.99–1.31)

To put the changes in overall disease trends compared to the composite control into some context, we estimated that the total hospitalised burden of pneumococcal pneumonia reduced by 4611 cases for all ages from April 2007 to March 2015, with large reductions in the 65+ years age group (1917 cases) and in children under 15 years of age (1315 cases). Cases of otitis media with tympanostomy reduced by 30,649, 83% of which (25,565) were in children aged < 15 years. We estimated a reduction of 19,712 cases for pneumonia of unspecified organism for the same age group, though this was counterbalanced by an estimated increase of 251,894 cases for the 65+ year age group. Further details of the estimated net change in the number of cases since April 2007 can be found in Table 4.

**Table 4:** The estimated net change in the total number of cases since April 2007 when comparing the observed relative incidence of each disease endpoint to the observed relative incidence of the composite control

Disease endpoint	<2 years	2–4	5–14	15–24	25–44	45–64	65+	Total
IPD	–3209	–729	–454	–637	–2202	–4107	–10,790	–22,128
Pneumococcal pneumonia	–854	–187	–274	–282	–588	–509	–1917	–4611
Pneumonia (unsp. organism)	–11,583	–2651	–5479	294	6067	38,109	251,894	276,652
Empyema	–291	–61	–412	–129	299	395	780	581
Lung abscess with pneumonia	–2	–14	20	–12	125	183	353	615
Non-specific sepsis	6500	1209	1277	876	4318	17,238	27,253	58,670
Pneumococcal sepsis	–1295	–152	–44	–31	–171	–80	–318	–2092
Otitis media	–7473	–2302	–17,584	1978	–2638	–5297	295	–37,568
Otitis media with tympanostomy	–6391	–3252	–15,922	–980	–1164	–2864	–77	–30,649

## Discussion

Our national study shows that the PCV programme in England has been associated with significant reductions in hospital admissions across a range of non-specific disease endpoints in the age groups targeted for vaccination. The endpoints chosen were ones in which the pneumococcus is likely to have a causative role, although the percentage contribution of pneumococcal infection to these syndromes, and the serotype distribution, cannot be directly determined.

This is the first study to investigate the impact of the PCV programme on hospital admissions for a wide range of pneumococcal-specific and syndromic disease endpoints across all ages in England. In addition, we sought to improve the interpretation of our impact analyses by comparing with changes in control conditions to allow for biases generated by secular trends in admission practice. We also used as a benchmark changes in overall IPD incidence, which given the specificity of this outcome is likely to represent a maximal impact when compared with less specific disease endpoints such as all-cause pneumonia.

The reduction in admissions for pneumonia of unspecified organism in children under 5 years of age (which comprises more than 90% of all pneumonia admissions in this age group) is similar to that in studies in Sweden [12, 13], Uruguay [14], Scotland [15] and southern Israel [16], where reductions ranging from 19 to 32% following the implementation of sequential PCV7/PCV13 programmes have been reported. The consistency of these observations in different settings suggests a causal association with PCV7/PCV13 use. The reductions observed in pneumonia admissions in vaccine-eligible children post-licensure are greater than suggested by the pre-licensure trial of PCV7 in the USA in which clinically diagnosed pneumonias were only reduced by 4.3% [17]. Unlike the US trial, our study was restricted to pneumonias requiring hospital admission. As the contribution of the pneumococcus to hospital-admitted pneumonias in children is likely to be higher than in those not admitted, the percentage reduction in inpatient pneumonia activity is likely to be higher than 4.3%. The greater than expected benefit of sequential PCV7/PCV13 programmes may also reflect the contribution of the additional serotypes in PCV13 to pneumonia — especially serotypes 1 and 19A. The development of the indirect effect will also increase the reduction observed in the vaccinated age groups.

Reductions in older age groups in pneumonia of unspecified origin could not be demonstrated, with increases in admissions for J18 observed in all age groups from 15 years upwards, continuing the trend observed before the introduction of the PCV7 programme in 2004 [18]. A similar increase in 65+ year olds post-PCV7/PCV13 implementation has also been reported from Scotland, accompanied by a reduction in the length of stay [15]. These changes in older age groups may represent an increasing trend towards short stay emergency hospital admissions in an aging population, as suggested by a National Audit Office report [19]. The increase in pneumonia admissions in our study persisted even after taking into account the upward trend in admissions for the composite control conditions (Additional file 1: Figure S3). However, the ratio of the IRR for J18 compared to the IRR for each individual control condition was closer to 1 when using urinary tract infections as the control in those aged 65 years and over (Fig. 3); admissions for UTIs in the elderly are likely to be subject to similar emergency admission practices as acute respiratory infections. Few other studies have assessed the indirect impact of PCV in older age groups. In the USA, a reduction in all-cause pneumonia admissions in 65+ year olds was reported in a study that encompassed post-PCV7 and PCV13 periods ranging from 7% in 65–74 year olds to 23% in 85+ year olds [20], but this could not be replicated in a recent analysis from Australia [21] or in our study. A hospital-based prospective study in England that used urinary antigen detection methods showed a reduction in admissions for non-bacteraemic vaccine-type pneumonia caused by a PCV13 serotype in 65+ year olds similar in magnitude to the reduction in IPD, confirming the indirect protection against non-bacteraemic pneumococcal pneumonia [22]. However, there was little reduction in overall pneumonia admissions, which may reflect serotype replacement, secular trends in non-vaccine serotypes or changes in the epidemiology of other non-pneumococcal pathogens.

We observed a significant reduction of 80% in pneumococcal pneumonia in children under 2 years of age (Table 2, IRR column) which was similar to the reduction in IPD. Since the diagnosis of pneumococcal pneumonia requires evidence of the causative organism, the correspondence between these two disease outcomes is to be expected. Reductions in pneumococcal pneumonia were also observed in other age groups consistent with the indirect protection observed for IPD [8]. For pneumococcal sepsis, which includes pneumococcal meningitis and arthritis, significant reductions were observed in children under 15 years, with the largest reduction (66%) in children under 2 years old. The incidence of pneumococcal sepsis when added to pneumococcal pneumonia was considerably lower than the incidence of laboratory-confirmed IPD in all age groups. This is consistent with a previous study in which we linked laboratory-confirmed IPD reports with HES admissions and found that only a minority of laboratory-reported IPD cases had a specific pneumococcal sepsis or pneumonia code in HES [23], thus highlighting the limitations in documenting pathogen-specific causes of admission in HES.

Our results for the impact on empyema (Fig. 1, Table 2) are comparable to those reported in the USA [24] and Scotland [25], where the incidence of empyema increased after the introduction of PCV7 but declined after PCV13 for children aged < 15 years. This suggests that empyema is linked to additional serotypes covered by PCV13 which increased due to serotype replacement post-PCV7 and is consistent with studies showing that the three most prevalent serotypes causing empyema are 3, 1 and 19A [23], all of which are covered by PCV13 but not PCV7.

Mixed results were obtained for the other non-specific disease endpoints. No reductions for non-specific sepsis were observed with increases both in raw IRRs and IRRs relative to the composite control in all age groups. This suggests that these codes do not contain many cases of occult pneumococcal sepsis, at least not with a predominance attributable to vaccine-type serotypes. With the exception of children less than 2 years old, the increase in incidence was greater for the risk groups than the non-risk group (Additional file 1: Figures S4-S6). The reasons for this difference are unclear, but they reflect our findings in Table 3 showing that the greatest increases in incidence for pneumonia were also in the risk groups. The findings reported from both a Finnish trial of PCV10 [9] and a post-licensure study [26] in which large reductions in non-specific sepsis codes were observed in vaccinated children and vaccine-eligible children respectively could not be confirmed in this post-licensure study of a sequential PCV7/PCV13 programme. Possible reasons for this are differences in coding practices between the two countries, in serotype distribution of pneumococcal-attributable sepsis, or in the contribution of non-pneumococcal pathogens to admissions with a non-specific sepsis code. The analyses from Finland excluded laboratory-confirmed IPD cases, but we were unable to do this due to a lack of common patient identifiers in our HES and laboratory-confirmed IPD surveillance datasets. However, removal of laboratory-confirmed IPD cases from admissions with a non-specific sepsis diagnosis should reduce the proportion of such cases attributable to the pneumococcus and thus the potential impact of PCV on this disease endpoint. For lung abscess with pneumonia, the rIRRs against the composite control and against each control condition individually were less than one in age groups under 15 years and similar to that for empyema.

We were unable to detect a large reduction in admissions for all otitis media diagnoses in infants, echoing the efficacy trials of PCV7 on all-cause otitis media in Finland [27] and in the USA [28]. For placement of tympanostomy tubes, which reflects recurrent or more serious otitis media, the US trial showed a higher efficacy of 20.1% (95% CI 1.5–35.2%), and late follow-up of the Finnish trial cohort to 5 years of age showed an efficacy of 39% (95% CI 4–61%) against tube placements [29]. However, further follow-up of the Finnish cohort showed no sustained efficacy between 6 and 13 years of age [30]. Although we observed reductions in the incidence of otitis media with tympanostomies in vaccine-targeted cohorts, reductions were also observed in the older age groups which are unlikely to be due to the PCV programme. Ear tube placements for otitis media are uncommon in adults (Additional file 1: Table S4), and our findings are therefore difficult to interpret.

Within the risk group analysis the composite control was essential to use as a benchmark, as shown by the increase in admissions for the unrelated control conditions in those with risk factors. This may be an artefact due to better recording of comorbid conditions in the available HES ICD codes, or a real increase due to changes in admission practice, but the comparison with the control conditions allowed adjustment for such secular trends. The significant reductions in pneumococcal pneumonia in high-risk individuals of all ages confirms the herd immunity benefit of the PCV programme in these vulnerable groups and that the additional benefit of direct vaccination is limited [31]. The increase in older individuals in pneumonia of unspecified organism shown in Table 2 was largely among those in a risk group (Table 3). This may reflect their greater susceptibility to develop pneumonia from less pathogenic serotypes that have replaced the PCV13 vaccine types in the nasopharynx as has been shown for IPD in high-risk children [32].

The use of additional ICD codes to identify the outcomes of interest should increase sensitivity, but it would likely be at the cost of reduced specificity. The contribution of additional true positive or false positive cases when the ICD-10 codes are expanded will likely vary with age group, disease outcome and time period, so the effect is unpredictable, as shown by our analyses. On balance we preferred to restrict our main analyses to outcomes of interest in the first diagnosis field, as this is intended to capture the primary reason for admission and so should maximise specificity.

Our method of assessing the impact of the PCV programme in England by comparing the IRR for the outcome of interest with that in control conditions has some advantages over other methods such as time-series analyses that have been used to assess the effect of PCV on pneumonia. The latter method does not take account of factors other than vaccination that may have resulted in secular changes in admission, and the results are sensitive to the model used to fit the pre-and post-intervention trend lines [33]. Another variation in the use of control conditions against which to assess the impact of PCV on pneumonia has recently been evaluated in a methodological study using hospitalisation data from five countries in the Americas [34]. In that study the authors derived a composite control by aggregating pre-vaccination data across 17 disparate ICD-10 chapters plus 20 additional conditions and then assigned weights to each to generate a "synthetic" composite control whose pre-vaccination trend best matched pre-vaccination pneumonia trends. The post-vaccination data from the weighted synthetic controls were then used as a counterfactual against which to assess the impact of PCV. The weights applied to each ICD chapter/condition varied substantially between the five countries and within each country by age. In some age groups, the results were particularly sensitive to the inclusion of specific codes in the synthetic control. Overall the results when using a country-specific synthetic control were somewhat heterogenous between countries, though, in common with our findings, there was no evidence of a decline in all-cause pneumonia admissions in older adults in any country. The synthetic control method is computationally demanding, especially when dealing with very large datasets such as the national HES dataset and does not take account of interventions apart from vaccination that may have affected the incidence of the control conditions. Furthermore, a split into subgroups such as our analysis of risk- and non-risk groups is harder to achieve. Our results demonstrate that individual comparisons between the studied diseases and the constituent conditions of the composite control are informative. For example, when using urinary tract infections as the control, the increase in admissions for pneumonia of unspecified organism in adults aged 65+ years was minimised (21% relative increase); both are common infections in this age group and are likely affected by the same underlying changes in admission practices. However, when compared to trends for fractures which showed little change in 65+ year olds over the same period (Additional file 1: Table S7), the relative increase was maximal (88% increase). Based on the knowledge of how admission or management practices have changed in an individual country, it may be more efficient to select control conditions that will be unaffected by the PCV programme but similarly affected by known changes in health service utilisation.

### Limitations

Although the HES database has been running prior to the start of the study period, we were unable to extract data for episodes prior to April 2004 because patient identification fields were not available in the data, precluding identification of repeat admissions in the same individual. We were therefore only able to assess the impact of PCV7 using 2 years' worth of data prior to its introduction mid-2006. Our results would be more robust if additional data prior to April 2004 were able to be linked to our existing dataset. The HES database does not include information on any laboratory testing conducted for each individual, so we were unable to confirm the aetiology of each case.

We identified risk groups using only the information available within the selected admissions, rather than examining prior admissions for individuals in the years before the outcome diagnoses. This may have resulted in a lower sensitivity for risk group identification. However, we performed a subanalysis (unpublished) where more information from the HES dataset was extracted from previous years on a patient-by-patient basis for a sample of 1000 patients. Despite being much more work, adding information from previous years increased the risk group identification only from just below 80% to just over 90%, which we deemed acceptable given the purpose of this analysis.

Another potential limitation is that we did not try to estimate the impact of PCV7 and PCV13 separately. We did this because our laboratory-confirmed IPD analyses showed that the impact of serotype replacement post-PCV7, particularly in relation to 19A and 7F, which offset some of the benefit of PCV7 was mitigated by the subsequent introduction of PCV13 [8], and thus it was more informative to focus on the impact of the sequential PCV7 and PCV13 programme as a whole than each separately.

Our analysis of the potential change in the case burden for each of the diagnoses of interest may have underestimated the reported changes in Table 4, as the initial period from September 2006 through April 2007 was missed due to the use of HES data years.

Our use of five control conditions is a novel approach in assessing the impact of a pneumococcal vaccination programme on hospitalised disease outcomes. Our analysis could be improved if we had used more than five control conditions and conducted a more comprehensive analysis of the effect of using as controls conditions that were likely or unlikely to be affected by similar changes in admission practices as the pneumococcal-attributable outcomes. However, we were unable to extract data for more than five control conditions due to resource limitations, and with data from only 2 pre-PCV years available to us in HES, we were unable to conduct further investigations into the similarities between the control conditions and the disease outcomes of interest beyond the age distributions of cases and not being subject to other public health interventions (see Additional file 1: Figures S1 and S2). Furthermore, we did not assess the seasonality of the control conditions. However, we minimised the impact of seasonality by averaging over a 24-month period and including the same months pre- and post-PCV (April to March) for both outcomes of interest and control conditions. Despite being restricted to five controls, our analysis is an improvement on impact analyses that use no controls or the use of a single control condition which can be sensitive to changes in secular trends unrelated to the introduction of the PCV programme and therefore a source of bias in programme impact estimates [34].

We have reported disease trends for many age groups and many diagnoses of interest, so it is possible that our results may include some IRR estimates for which the upper 95% confidence is less than one by chance. However, a formal correction for multiple comparisons is not straightforward in this instance given the method that we have used. Furthermore use of only five controls in deriving rIRRs precluded any formal statistical comparison such as computation of confidence intervals, so uncertainty in this measure could only be depicted by showing maximum and minimum rIRR values across the control conditions.

It is also important to note that the overall burden of non-invasive pneumococcal disease will be determined not only by the impact of the PCV programme on disease caused by the pneumococcus but also by the epidemiology of other causative pathogens for pneumonia. Changes in the latter may mask an overall reduction in pneumococcal-attributable non-invasive disease, but this cannot be assessed by the type of impact assessment conducted here.

## Conclusions

By comparing the incidence of pneumococcal outcomes with unrelated conditions, and stratifying by risk group status, our method can help control for the effect of unrelated secular trends in admissions and help elucidate the impact of PCV in vulnerable population groups. Since the beginning the PCV programme, we observed a substantial reduction in all age groups for the hospitalised burden of pneumococcal pneumonia; for children under 15 we observe a decline in pneumonia with unspecified causative organism and pneumococcal sepsis, and for those under 2 years a reduction in otitis media with tympanostomies. For some age groups we observed increases in the disease burden which could be non PCV-related or due to replacing serotypes.

## Additional file

Additional file 1: Identification of cases, comorbidities and control conditions; additional analyses of the incidence of cases and control conditions; sensitivity analysis; further analyses on the increased incidence of non-specific sepsis. (DOCX 260 kb)

## Abbreviations

HES: Hospital Episode Statistics
ICD-10: International Statistical Classification of Diseases and Related Health Problems, 10th revision
IPD: Invasive pneumococcal disease
IRR: Incidence rate ratio
NHS: National Health Service
PCV: Pneumococcal conjugate vaccine
PCV10: 10-valent pneumococcal conjugate vaccine
PCV13: 13-valent pneumococcal conjugate vaccine
PCV7: seven-valent pneumococcal conjugate vaccine
PPV23: 23-valent pneumococcal polysaccharide vaccine
rIRR: Ratio of incidence rate ratios
