# Supplementary material for: Elucidating the impact of the pneumococcal conjugate vaccine programme on pneumonia, sepsis and otitis media hospital admissions in England using a composite control
Source: BMC Med. 2018 Feb 8;16:13. doi: 10.1186/s12916-018-1004-z (PMC5804014; doi:10.1186/s12916-018-1004-z)
Supplement: Additional file 1: — Identification of cases, comorbidities and control conditions; additional analyses of the incidence of cases and control conditions; sensitivity analysis; further analyses on the increased incidence of non-specific sepsis. (DOCX 260 kb) [file 12916_2018_1004_MOESM1_ESM.docx]

# Additional file 1

## Identification of cases, comorbidities and control conditions

### Identification of cases

| ICD-10 code | Diagnosis |
| --- | --- |
| **Pneumonia and** |  |
| **other respiratory conditions** |  |
| J13 | Pneumonia due to Streptococcus pneumoniae |
| J18 | Pneumonia: unspecified causative organism |
| J869 | Empyema |
| J851 | Abscess of lung with pneumonia |
|  |  |
| **Sepsis** |  |
| A403 | Sepsis due to Streptococcus pneumoniae |
| A409 | Streptococcal sepsis, unspecified |
| A419 | Sepsis, unspecified organism |
| A491 | Streptococcal and enterococcal infection, unspecified site |
| A499 | Other bacterial infection, unspecified |
| B953 | Streptococcus pneumoniae as the cause of diseases classified elsewhere |
| B955 | Unspecified streptococcus as the cause of diseases classified elsewhere |
| G001 | Pneumococcal meningitis |
| G009 | Bacterial meningitis, unspecified |
| M001 | Pneumococcal arthritis and polyarthritis |
| M009 | Pyogenic arthritis, unspecified |
| I301 | Infective pericarditis |
|  |  |
| **Otitis Media** |  |
| H65 | Nonsuppurative otitis media |
| H66 | Suppurative and unspecified otitis media |
| H67 | Otitis media in diseases classified elsewhere |

***Table S1****: ICD-10 codes used to identify cases of all disease endpoints.*

### Identification of comorbidities found to increase the risk of pneumococcal infection

| Risk group | ICD-10 codes |
| --- | --- |
| Chronic respiratory disease | J40, J41, J42, J43, J44, J47, J6, J7, J80, J81, J82, J83, J84, Q30, Q31, Q32, Q33, Q34, Q35, Q36, Q37 |
| Chronic heart disease | I05, I06, I07, I08, I09, I11, I12, I13, I20, I21, I22, I25, I27, I28, I3, I40, I41, I42, I43, I44, I45, I47, I48, I49, I50, I51, I52, Q2 |
| Chronic kidney disease | N00, N01, N02, N03, N04, N05, N07, N08, N11, N12, N14, N15, N16, N18, N19, N25, Q60, Q61 |
| Chronic liver disease | K70, K71, K72, K73, K74, K75, K76, K77, P788, Q44 |
| Diabetes | E10, E11, E12, E13, E14, E24, G590, G632, G730, G990, N083, O24, P700, P701, P702 |
| Immunosuppression | C81, C82, C83, C84, C85, C88, C90, C91, C92, C93, C94, C95, C96, B20, B21, B22, B23, B24, Z94, Z85, D561, D57, D61, D70, D71, D72, D73, D76, D80, D81, D82, D83, D84, K900 |
| Asplenia or dysfunction of the spleen | D73, D561, D578, D570, D571, K900 |
| Individuals with cochlear implants | Z962 |
| Individuals with cerebrospinal fluid leaks | G960 |

***Table S2****: ICD-10 codes used to identify risk groups.*

### Identification of the control conditions

| ICD-10 code | Diagnosis |
| --- | --- |
| **Urinary Tract Infections** |  |
| N10 | Acute tubulo-interstitial nephritis |
| N30 | Cystisis |
| N390 | Urinary tract infection, site not specified |
|  |  |
| **Skin conditions** |  |
| L00 | Staphylococcal scalded skin syndrome |
| L01 | Impetigo |
| L02 | Cutaneous abscess, furuncle and carbuncle |
| L03 | Cellulitis |
| L04 | Acute lymphadenitis |
| L05 | Pilonidal cyst |
| L08 | Other local infections of skin and subcutaneous tissue |
|  |  |
| **Fractures** |  |
| S02 | Fracture of skull and facial bones |
| S12 | Fracture of neck |
| S22 | Fracture of rib(s), sternum and thoracic spine |
| S32 | Fracture of lumbar spine and pelvis |
| S42 | Fracture of shoulder and upper arm |
| S52 | Fracture of forearm |
| S62 | Fracture at wrist and hand level |
| S72 | Fracture of femur |
| S82 | Fracture of lower leg, including ankle |
| S92 | Fracture of foot, except ankle |
|  |  |
| **Blood disorders** |  |
| D50-D53 | Nutritional anaemias |
| D55-D59 | Haemolytic anaemias |
| D60-D64 | Aplastic and other anaemias |
| D65-D69 | Coagulation defects, purpura and other haemorrhagic conditions |
| D70-D77 | Other diseases of blood and blood-forming organs |
| D80-D89 | Certain disorders involving the immune mechanism |
|  |  |
| **Disorders of the thyroid gland** |  |
| E00 | Congenital iodine-deficiency syndrome |
| E01 | Iodine-deficiency-related thyroid disorders and allied conditions |
| E02 | Subclinical iodine-deficiency hypothyroidism |
| E03 | Other hypothyroidism |
| E04 | Other nontoxic goitre |
| E05 | Thyrotoxicosis [hyperthyroidism] |
| E06 | Thyroiditis |
| E07 | Other disorders of thyroid |

***Table S3****: ICD-10 codes for all control conditions.*

## Incidence of the control conditions in the pre-PCV era


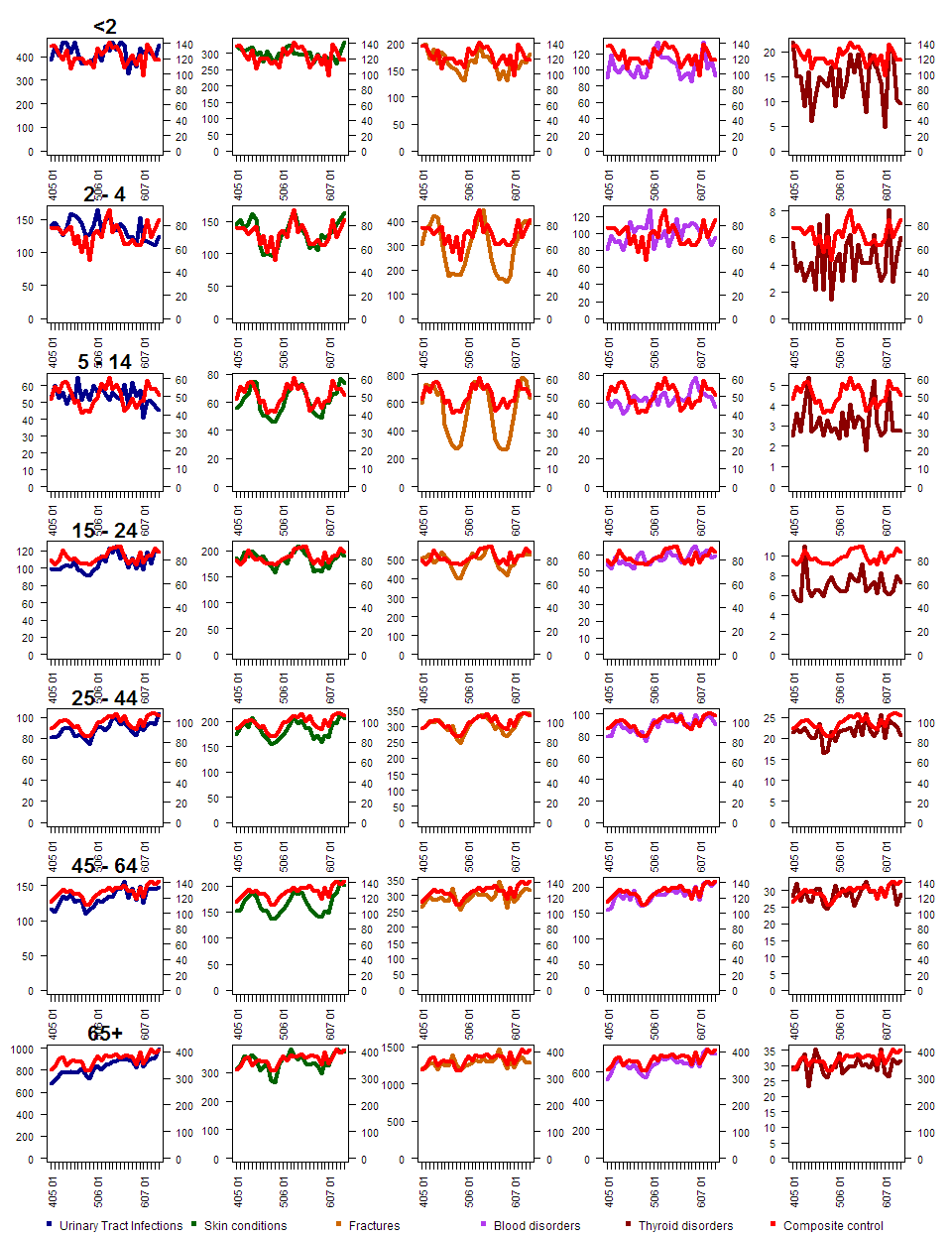


***Figure S1****: Incidence per 100,000 for each control condition and the composite control before the introduction of the PCV programme in September 2006. The x-axes refer to the financial year and month for the pre-PCV programme period starting from April 2004 (405 01) to August 2006 (607 05).*

## Incidence of the control conditions in the pre-PCV era compared to pneumonia of unspecified causative organism


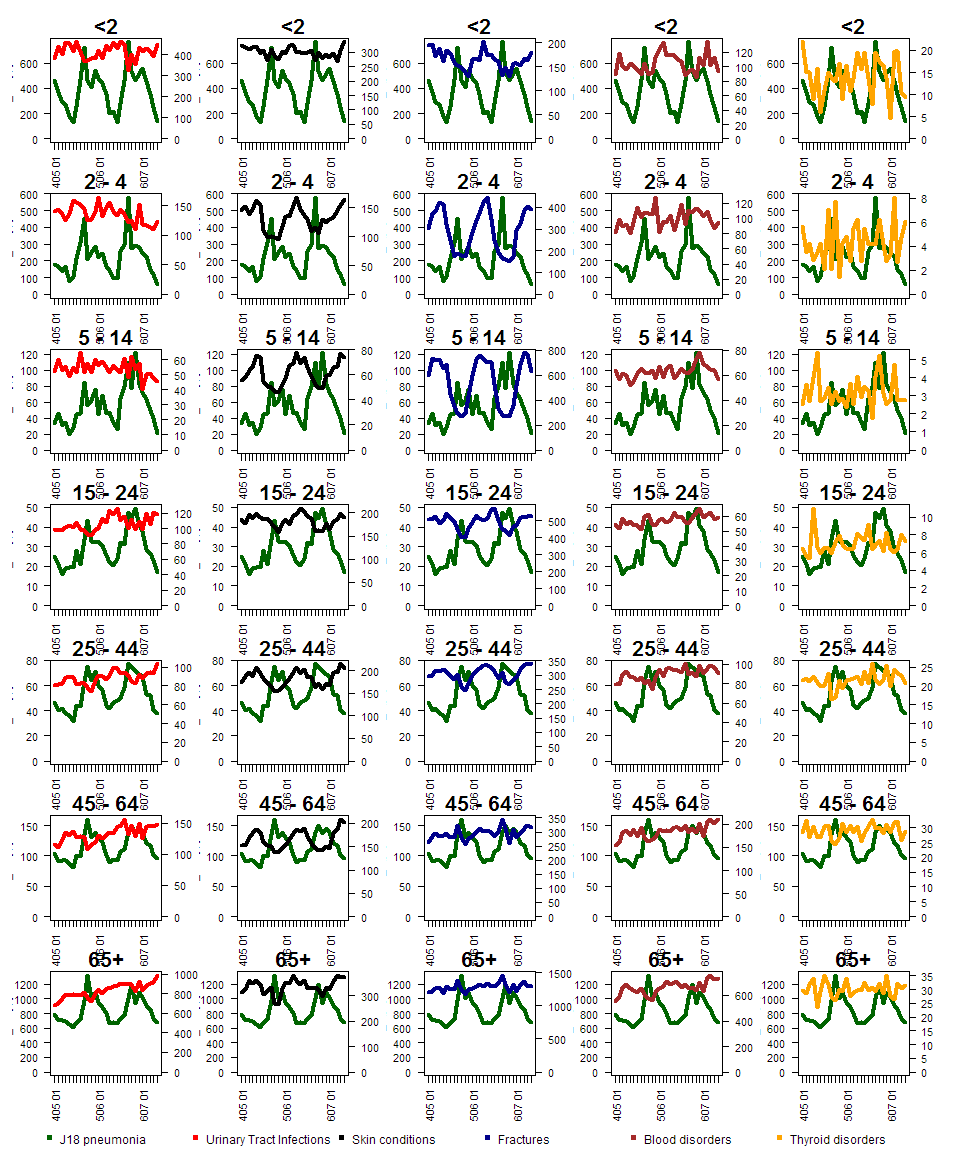


***Figure S2****: Incidence per 100,000 for each control condition and pneumonia of unspecified causative organism before the introduction of the PCV programme in September 2006. The x-axes refer to the financial year and month for the pre-PCV programme period starting from April 2004 (405 01) to August 2006 (607 05).*

## Case numbers by age

| ICD-10 | Total | <2 yrs | 2 - 4 | 5 - 14 | 15 - 24 | 25 - 44 | 45 - 64 | 65+ |
| --- | --- | --- | --- | --- | --- | --- | --- | --- |
| **Pneumonia** |  |  |  |  |  |  |  |  |
| J13 | 30,459 | 719 | 606 | 610 | 927 | 4,709 | 7,833 | 14,523 |
| J18 | 1,683,478 | 46,823 | 43,035 | 33,720 | 24,651 | 102,447 | 238,240 | 1,188,268 |
|  |  |  |  |  |  |  |  |  |
| **Other respiratory conditions** |  |  |  |  |  |  |  |  |
| J869 | 23,434 | 667 | 1,130 | 1,367 | 977 | 3,985 | 6,928 | 8,253 |
| J851 | 2,616 | 50 | 58 | 54 | 85 | 511 | 779 | 1,057 |
|  |  |  |  |  |  |  |  |  |
| **Sepsis** |  |  |  |  |  |  |  |  |
| Any sepsis | 304,714 | 17,307 | 3,96 | 5,000 | 7,237 | 25,014 | 61,238 | 183,363 |
| Any non-specific sepsis | 296,738 | 15,759 | 3,613 | 4,660 | 7,026 | 24,067 | 59,295 | 180,823 |
| Any pneumococcal sepsis | 7,976 | 1,548 | 351 | 340 | 211 | 947 | 1,943 | 2,540 |
| A403 | 3,672 | 509 | 169 | 120 | 82 | 370 | 756 | 1,601 |
| A409 | 3,400 | 181 | 90 | 94 | 73 | 333 | 615 | 1,982 |
| A419 | 243,281 | 11,925 | 1,803 | 2,025 | 3,731 | 15,158 | 47,742 | 159,791 |
| A491 | 3,704 | 406 | 521 | 561 | 146 | 334 | 488 | 1,225 |
| A499 | 5,833 | 518 | 245 | 234 | 225 | 533 | 1,048 | 2,997 |
| B953 | 9 | 1 | 0 | 0 | 0 | 4 | 0 | 4 |
| B955 | 10 | 0 | 0 | 2 | 0 | 4 | 0 | 4 |
| G001 | 4,188 | 1,045 | 177 | 218 | 131 | 570 | 1,150 | 867 |
| G009 | 4,676 | 1,078 | 125 | 226 | 724 | 1,116 | 765 | 603 |
| M001 | 173 | 25 | 9 | 7 | 7 | 45 | 79 | 1 |
| M009 | 33,375 | 1,760 | 849 | 1,535 | 1,424 | 5,238 | 7,889 | 14,442 |
| I301 | 3,821 | 10 | 10 | 18 | 728 | 1,484 | 1,070 | 477 |
|  |  |  |  |  |  |  |  |  |
| **Otitis Media** |  |  |  |  |  |  |  |  |
| All otitis media | 408,999 | 38,763 | 105,549 | 163,169 | 15,542 | 28,751 | 34,482 | 22,604 |
| OM, tympanostomy | 315,908 | 14,694 | 89,650 | 145,343 | 8,926 | 17,235 | 23,703 | 16,298 |
| H65 | 301,981 | 13,962 | 84,649 | 138,347 | 8,682 | 16,842 | 23,301 | 16,128 |
| H66 | 108,598 | 25,135 | 21,396 | 25,282 | 6,904 | 12,017 | 11,264 | 6,531 |
| H67 | 20 | 13 | 0 | 2 | 0 | 1 | 2 | 2 |

***Table S4****: The number of cases of pneumonia, sepsis and otitis media in England, split by age group from April 2004 through March 2015.*

## Cases of multiple disease endpoints

| Disease | Pneumonia/Other resp. | Sepsis | Otitis media |
| --- | --- | --- | --- |
| J13 pneumonia | n/a | 3,849 (0.53%) | 81 (0.01%) |
| J18 pneumonia | n/a | 117,473 (16.10%) | 2,230 (0.41%) |
| Other respiratory | n/a | 3,557 (0.49%) | 44 (0.01%) |
| **Any sepsis** | 44,259 (3.58%) | n/a | 1,947 (0.36%) |
| Non-specific sepsis | 39,704 (3.21%) | n/a | 475 (0.09%) |
| Pneumococcal sepsis | 4,752 (0.38%) | n/a | 882 (0.16%) |
| **Otitis media** | 1,139(0.09%) | 1,952 (0.27%) | n/a |

***Table S5****: Concurrent diagnoses for patients with pneumonia and other respiratory conditions, sepsis or otitis media.*

## Expanding the number of ICD-10 fields used to identify cases to 3 and 20

| Disease endpoint | Geometric mean ratio of IRRs (range: min-max) 20 codes | Geometric mean ratio of IRRs (range: min-max) 3 codes | Geometric mean ratio of IRRs (range: min-max) 1 code |
| --- | --- | --- | --- |
| **<2 years** |  |  |  |
| Pneumococcal pneumonia | 0.30 (0.23-0.40) | 0.28 (0.22-0.38) | 0.19 (0.15-0.25) |
| Pneumonia (unsp. organism) | 0.76 (0.59-1.02) | 0.74 (0.57-0.98) | 0.66 (0.51-0.89) |
| Empyema | 0.57 (0.44-0.76) | 0.53 (0.41-0.71) | 0.44 (0.34-0.59) |
| Lung abscess with pneumonia | 0.73 (0.56-0.98) | 0.64 (0.49-0.85) | 0.49 (0.38-0.65) |
| Non-specific sepsis | 2.30 (1.77-3.07) | 2.35 (1.81-3.14) | 2.79 (2.15-3.73) |
| Pneumococcal sepsis | 0.64 (0.50-0.86) | 0.56 (0.43-0.75) | 0.33 (0.25-0.44) |
| Otitis media | 0.86 (0.66-1.15) | 0.84 (0.65-1.12) | 0.76 (0.58-1.01) |
| Otitis media with tympanostomy | 0.55 (0.43-0.74) | 0.55 (0.42-0.74) | 0.51 (0.39-0.69) |
|  |  |  |  |
| **2 - 4 years** |  |  |  |
| Pneumococcal pneumonia | 0.54 (0.42-0.75) | 0.53 (0.41-0.74) | 0.47 (0.36-0.65) |
| Pneumonia (unsp. organism) | 0.85 (0.65-1.18) | 0.83 (0.64-1.15) | 0.80 (0.62-1.11) |
| Empyema | 0.69 (0.53-0.95) | 0.66 (0.51-0.91) | 0.57 (0.44-0.79) |
| Lung abscess with pneumonia | 0.65 (0.50-0.90) | 0.59 (0.46-0.82) | 0.70 (0.54-0.98) |
| Non-specific sepsis | 1.94 (1.49-2.69) | 1.97 (1.51-2.73) | 2.54 (1.95-3.52) |
| Pneumococcal sepsis | 1.03 (0.79-1.43) | 0.97 (0.74-1.34) | 0.53 (0.41-0.74) |
| Otitis media | 0.97 (0.74-1.34) | 0.95 (0.73-1.32) | 0.92 (0.70-1.27) |
| Otitis media with tympanostomy | 0.89 (0.69-1.24) | 0.89 (0.68-1.23) | 0.88 (0.68-1.22) |
|  |  |  |  |
| **5 - 14 years** |  |  |  |
| Pneumococcal pneumonia | 0.38 (0.33-0.49) | 0.37 (0.32-0.48) | 0.31 (0.27-0.41) |
| Pneumonia (unsp. organism) | 0.76 (0.66-0.98) | 0.73 (0.64-0.95) | 0.67 (0.59-0.87) |
| Empyema | 0.48 (0.42-0.63) | 0.46 (0.40-0.60) | 0.43 (0.37-0.56) |
| Lung abscess with pneumonia | 0.64 (0.56-0.84) | 0.54 (0.47-0.70) | 0.42 (0.37-0.55) |
| Non-specific sepsis | 1.55 (1.35-2.02) | 1.56 (1.36-2.03) | 2.02 (1.76-2.63) |
| Pneumococcal sepsis | 1.26 (1.10-1.64) | 1.20 (1.05-1.57) | 0.73 (0.64-0.96) |
| Otitis media | 0.77 (0.67-1.00) | 0.76 (0.66-0.99) | 0.74 (0.64-0.96) |
| Otitis media with tympanostomy | 0.73 (0.64-0.95) | 0.73 (0.64-0.95) | 0.72 (0.63-0.94) |
|  |  |  |  |
| **15 - 24 years** |  |  |  |
| Pneumococcal pneumonia | 0.55 (0.45-0.76) | 0.53 (0.43-0.73) | 0.50 (0.40-0.69) |
| Pneumonia (unsp. organism) | 1.29 (1.04-1.78) | 1.19 (0.96-1.64) | 1.04 (0.84-1.43) |
| Empyema | 0.80 (0.65-1.11) | 0.76 (0.61-1.05) | 0.79 (0.64-1.09) |
| Lung abscess with pneumonia | 0.95 (0.77-1.31) | 0.93 (0.75-1.28) | 1.01 (0.82-1.39) |
| Non-specific sepsis | 1.31 (1.06-1.81) | 1.34 (1.08-1.84) | 1.65 (1.33-2.27) |
| Pneumococcal sepsis | 0.80 (0.65-1.11) | 0.84 (0.68-1.16) | 0.74 (0.60-1.03) |
| Otitis media | 0.85 (0.69-1.18) | 0.82 (0.67-1.14) | 0.75 (0.61-1.04) |
| Otitis media with tympanostomy | 0.77 (0.62-1.06) | 0.76 (0.62-1.05) | 0.75 (0.61-1.04) |
|  |  |  |  |
| **25 - 44 years** |  |  |  |
| Pneumococcal pneumonia | 0.69 (0.53-0.83) | 0.67 (0.51-0.81) | 0.68 (0.52-0.82) |
| Pneumonia (unsp. organism) | 1.35 (1.04-1.62) | 1.24 (0.95-1.49) | 1.11 (0.86-1.34) |
| Empyema | 1.11 (0.85-1.34) | 1.08 (0.83-1.30) | 1.11 (0.85-1.33) |
| Lung abscess with pneumonia | 1.81 (1.39-2.18) | 1.71 (1.31-2.06) | 1.74 (1.34-2.10) |
| Non-specific sepsis | 1.35 (1.04-1.63) | 1.39 (1.07-1.68) | 1.78 (1.37-2.15) |
| Pneumococcal sepsis | 0.90 (0.69-1.08) | 0.83 (0.64-1.00) | 0.70 (0.54-0.84) |
| Otitis media | 0.91 (0.70-1.10) | 0.88 (0.68-1.06) | 0.82 (0.63-0.98) |
| Otitis media with tympanostomy | 0.82 (0.63-0.99) | 0.82 (0.63-0.98) | 0.81 (0.62-0.98) |
|  |  |  |  |
| **45 - 64 years** |  |  |  |
| Pneumococcal pneumonia | 0.89 (0.68-1.01) | 0.88 (0.67-1.00) | 0.91 (0.69-1.03) |
| Pneumonia (unsp. organism) | 1.57 (1.19-1.78) | 1.47 (1.12-1.67) | 1.44 (1.09-1.63) |
| Empyema | 1.08 (0.82-1.23) | 1.03 (0.78-1.17) | 1.07 (0.81-1.21) |
| Lung abscess with pneumonia | 1.77 (1.34-2.00) | 1.72 (1.30-1.95) | 1.61 (1.22-1.83) |
| Non-specific sepsis | 1.42 (1.08-1.61) | 1.50 (1.13-1.70) | 2.28 (1.73-2.58) |
| Pneumococcal sepsis | 1.02 (0.77-1.16) | 0.92 (0.70-1.05) | 0.89 (0.67-1.01) |
| Otitis media | 0.81 (0.62-0.92) | 0.78 (0.59-0.89) | 0.74 (0.56-0.83) |
| Otitis media with tympanostomy | 0.76 (0.58-0.86) | 0.76 (0.58-0.86) | 0.75 (0.57-0.86) |
|  |  |  |  |
| **65+ years** |  |  |  |
| Pneumococcal pneumonia | 0.75 (0.57-0.89) | 0.77 (0.59-0.91) | 0.87 (0.66-1.03) |
| Pneumonia (unsp. organism) | 1.53 (1.17-1.82) | 1.51 (1.15-1.79) | 1.58 (1.21-1.88) |
| Empyema | 1.27 (0.97-1.50) | 1.17 (0.89-1.39) | 1.20 (0.92-1.43) |
| Lung abscess with pneumonia | 1.69 (1.29-2.01) | 1.75 (1.33-2.08) | 1.96 (1.50-2.33) |
| Non-specific sepsis | 1.15 (0.88-1.37) | 1.19 (0.90-1.41) | 1.66 (1.26-1.97) |
| Pneumococcal sepsis | 0.90 (0.68-1.06) | 0.86 (0.65-1.02) | 0.86 (0.66-1.02) |
| Otitis media | 1.00 (0.76-1.19) | 0.94 (0.72-1.12) | 0.90 (0.69-1.07) |
| Otitis media with tympanostomy | 0.90 (0.69-1.07) | 0.90 (0.69-1.07) | 0.90 (0.69-1.07) |

***Table S6****: Comparing the estimated geometric mean ratios of incidence rate ratios for each disease endpoint relative to the composite control (first ICD-10 code only) when identifying eligible admissions by using 20 and 3 ICD-10 diagnosis codes.*

## Pneumonia of unspecified causative organism in the elderly


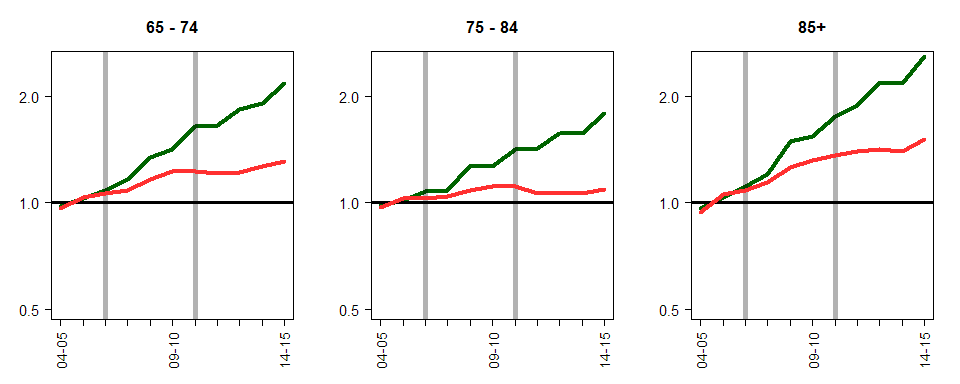


***Figure S3****: The relative incidence of pneumonia with unknown causative organism (green) plotted with the relative incidence of the composite control (red). The relative incidence is plotted on a log scale.*

## Investigating the increased incidence of non-specific sepsis

Note that the identification of risk-groups for the <2 age group is problematic - the original analysis that identified comorbidities to flag for risk-groups was unable to report results for this age group and therefore charts displaying the relative incidence of pneumococcal diseases for risk-groups in the <2 should be viewed cautiously.


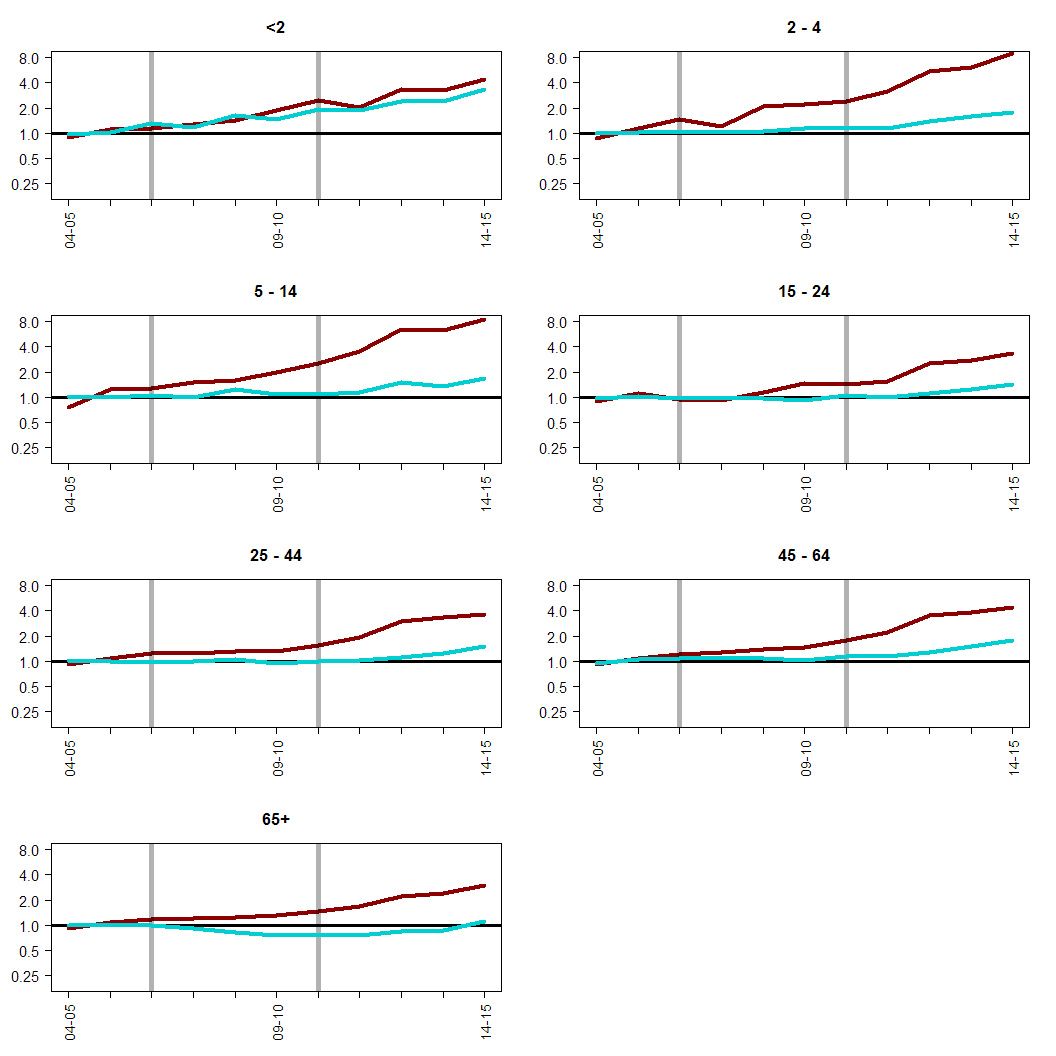


***Figure S4****: The relative incidence in at-risk (dark red) and non-risk (light blue) groups of all non-specific sepsis, calculated by taking the incidence per 100,000 reported in the pre-PCV era (1st April 2004 - 31st March 2006) as the denominator. The relative incidence is plotted on a log scale.*

### The relative incidence of sepsis of unspecified organism (ICD-10: A419)


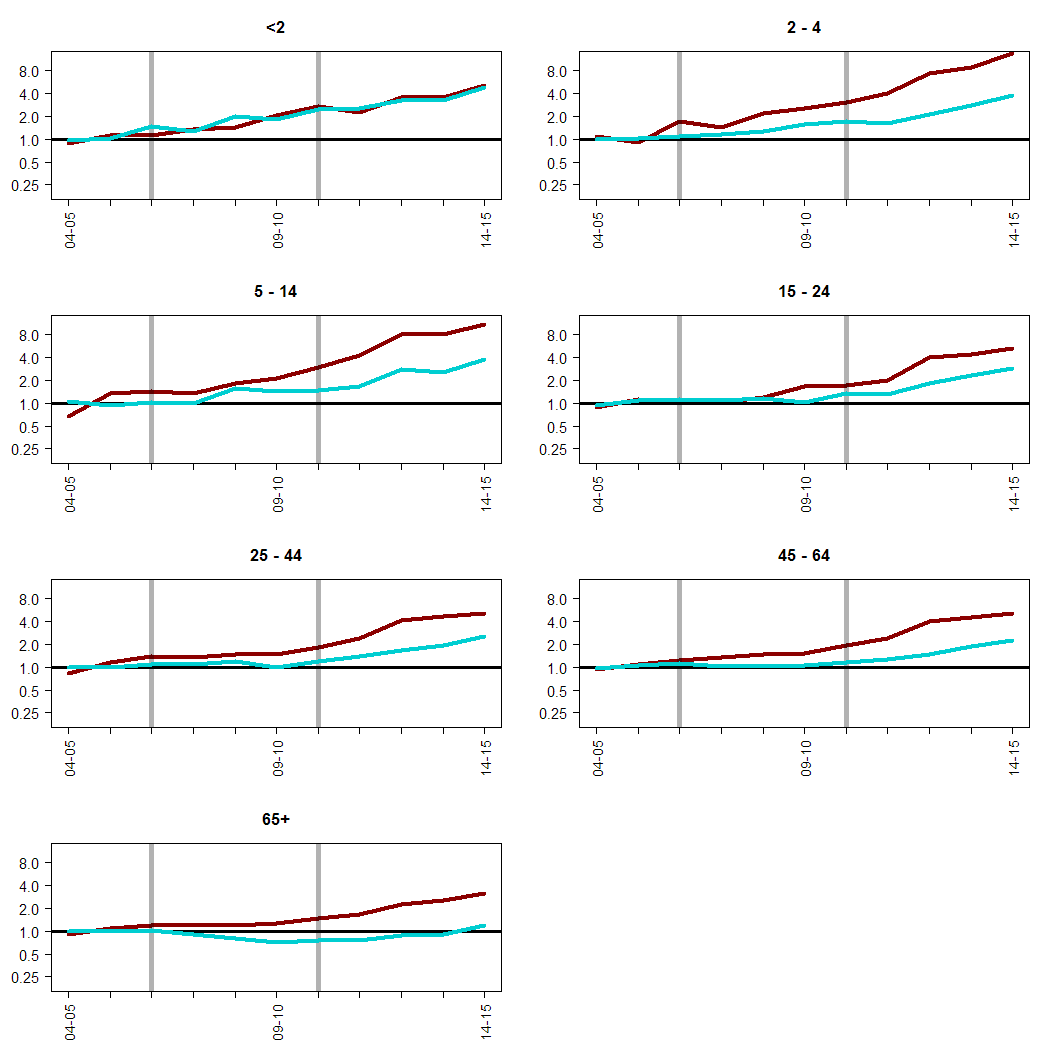


***Figure S5****: The relative incidence in at-risk (dark red) and non-risk (light blue) groups of sepsis of unspecified organism (ICD-10: A419), calculated by taking the incidence per 100,000 reported in the pre-PCV era (1st April 2004 - 31st March 2006) as the denominator. The relative incidence is plotted on a log scale.*

### The relative incidence of non-specific sepsis that is not identified as "sepsis of unspecified organism" (ICD-10s: A409, A491, A499, B955, G009, M009, I301)


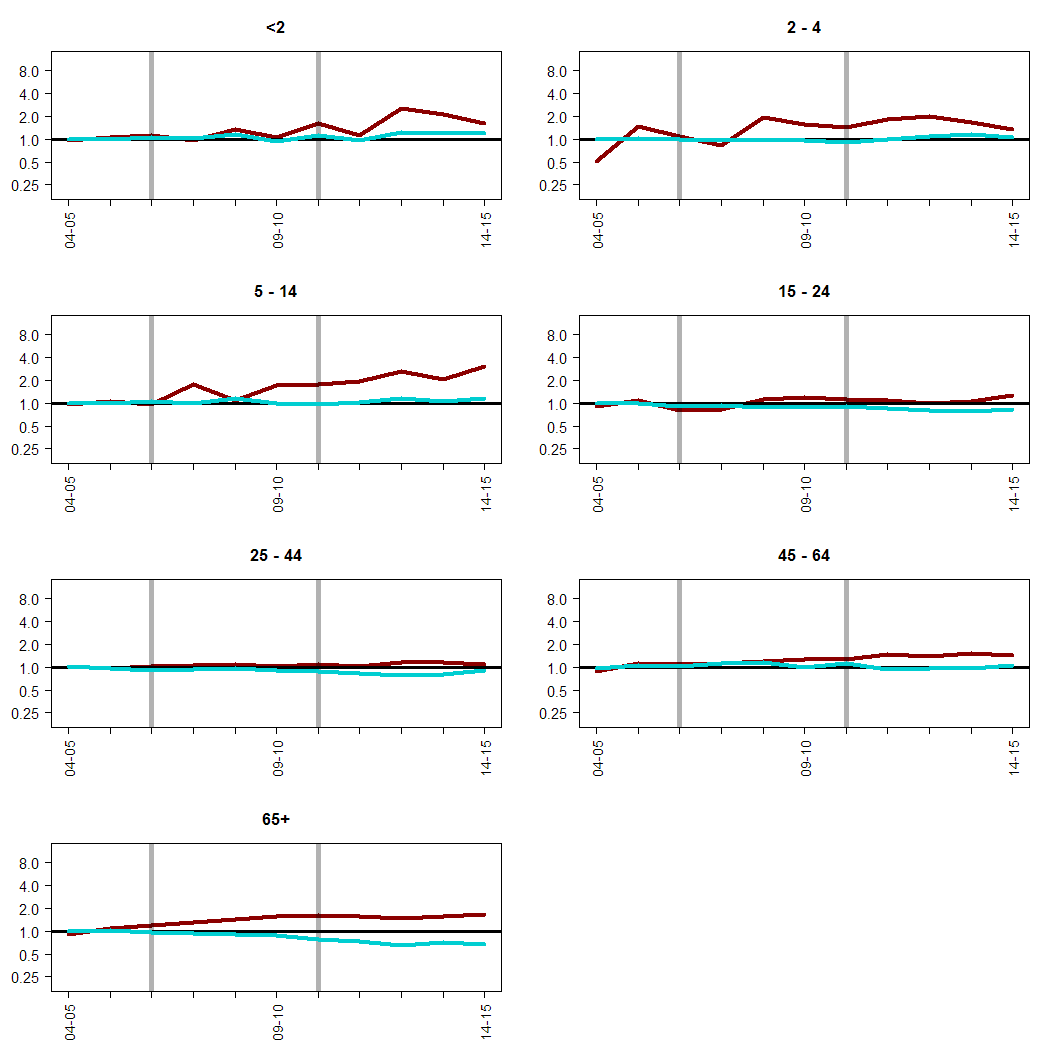


***Figure S6****: The relative incidence in at risk (dark red) and non-risk (light blue) groups of non-specific sepsis that is not identified as "sepsis of unspecified organism" (ICD-10s: A409, A491, A499, B955, G009, M009, I301), calculated by taking the incidence per 100,000 reported in the pre-PCV era (1st April 2004 - 31st March 2006) as the denominator. The relative incidence is plotted on a log scale.*

## Incidence rate ratios for each control condition

| Disease IRRs (95% CI) | UTI | Skin | Fractures | Blood | Thyroid | IPD |
| --- | --- | --- | --- | --- | --- | --- |
| **<2 years** | 1.06 (1.04-1.09) | 0.77 (0.75-0.80) | 0.99 (0.95-1.03) | 1.08 (1.03-1.13) | 1.34 (1.17-1.47) | 0.28 (0.25-0.32) |
| **2 - 4 years** | 0.79 (0.76-0.82) | 1.01 (0.97-1.05) | 0.96 (0.93-0.98) | 1.17 (1.12-1.22) | 0.65 (0.51-0.78) | 0.29 (0.24-0.34) |
| **5 - 14 years** | 1.04 (1.01-1.07) | 1.15 (1.12-1.19) | 0.83 (0.82-0.84) | 1.24 (1.20-1.28) | 1.20 (1.05-1.31) | 0.27 (0.23-0.31) |
| **15 - 24 years** | 1.25 (1.23-1.28) | 1.08 (1.06-1.09) | 0.83 (0.82-0.84) | 1.41 (1.37-1.44) | 1.22 (1.12-1.29) | 0.29 (0.25-0.34) |
| **25 - 44 years** | 1.24 (1.22-1.26) | 1.09 (1.08-1.10) | 1.00 (0.99-1.01) | 1.56 (1.54-1.58) | 1.19 (1.15-1.22) | 0.35 (0.32-0.37) |
| **45 - 64 years** | 1.38 (1.36-1.40) | 1.23 (1.21-1.24) | 1.17 (1.16-1.18) | 1.74 (1.72-1.76) | 1.16 (1.13-1.19) | 0.50 (0.48-0.53) |
| **65+ years** | 1.63 (1.62-1.64) | 1.16 (1.15-1.17) | 1.05 (1.04-1.05) | 1.40 (1.39-1.41) | 1.07 (1.03-1.10) | 0.66 (0.63-0.69) |

***Table S7****: Incidence rate ratios for each control condition and IPD.*

## Comparing the relative incidence of each disease endpoint to the composite control


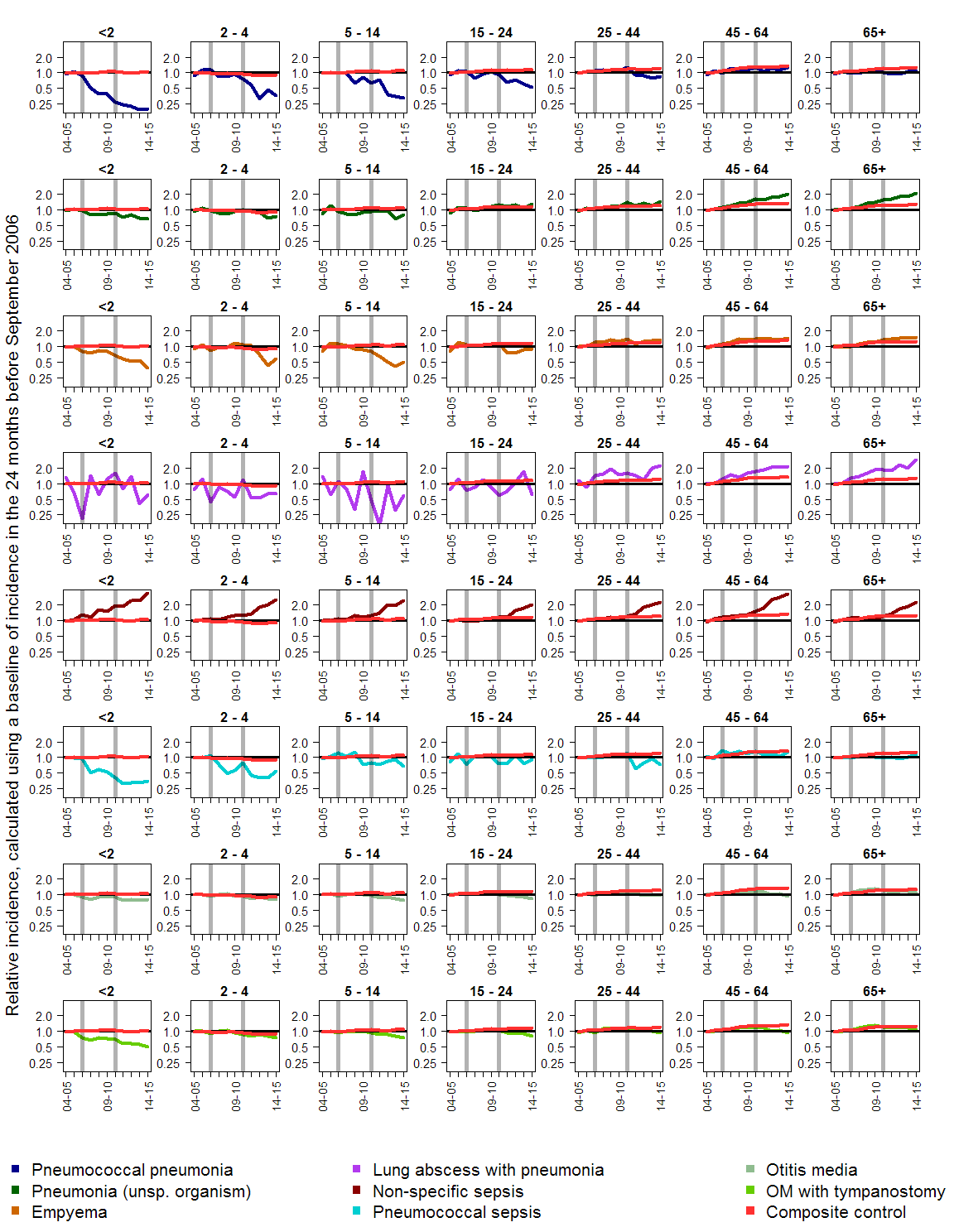


***Figure S7****: The relative incidence of all disease endpoints, calculated by taking the incidence per 100,000 reported in the pre-PCV era (1st April 2004 - 31st March 2006) as the denominator. The relative incidence is plotted on a log scale.*

## Calculation of incidence rate ratios and 95% confidence intervals

### Incidence rate ratios

The incidence rate ratios for disease endpoints was calculated using the following equation:

$$IRR=\frac{\frac{C_{post}}{T_{post}}}{\frac{C_{pre}}{T_{pre}}}$$

with $C_{post}$ and $C_{pre}$ as the number of cases post-PCV and pre-PCV respectively, and $T_{post}$ and $T_{pre}$ as each time period (person-years) during which those cases occurred.

### IRR confidence intervals

The endpoints of the 95% confidence intervals of incidence rate ratios was calculated using the following equation:

$$\exp\left( ln(IRR)\pm1.96\sqrt{\frac{1}{C_{pre}}+\frac{1}{C_{post}}} \right)$$

### Ratio of IRRs using the composite controls and range

The ratio of incidence rate ratios and range were calculated using the following equations:

$$rIRR^{5}=\prod_{i=1}^{5} IRR_{j}$$

and

$$range_{min}=minimum\{IRR_{j}\}$$

$$range_{max}=maximum\{IRR_{j}\}$$

where *j* $\epsilon$ *{1,2,3,4,5}* for all five control conditions.

## Using different post-PCV13 periods

### Case ratios

#### Pneumococcal pneumonia


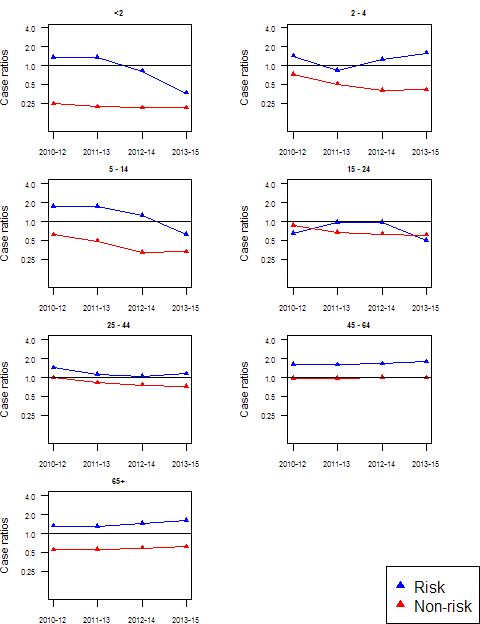


***Figure S8****: Estimated age-specific case ratios for pneumococcal pneumonia split by risk group. The denominator refers to cases for the pre-PCV era (1st April 2004 - 31st March 2006) and post-PCV era (1st April 2013 - 31st March 2015), and the numerator is varied using two financial years since the introduction of PCV13. The case ratios are plotted on a log scale.*

#### Pneumonia of unspecified causative organism


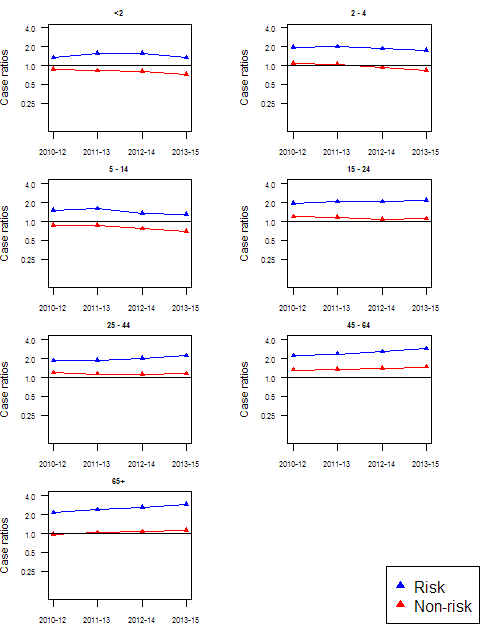


***Figure S9****: Estimated age-specific case ratios for pneumonia of unspecified causative organism split by risk group. The denominator refers to cases for the pre-PCV era (1st April 2004 - 31st March 2006) and post-PCV era (1st April 2013 - 31st March 2015), and the numerator is varied using two financial years since the introduction of PCV13. The case ratios are plotted on a log scale.*

### Ratio of case ratios

#### Pneumococcal pneumonia


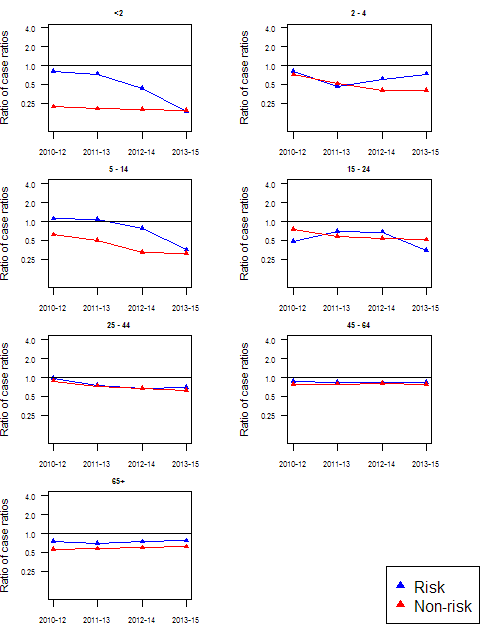


***Figure S10****: Estimated age-specific ratios of case ratios for pneumococcal pneumonia split by risk group. The denominator refers to cases for the pre-PCV era (1st April 2004 - 31st March 2006) and post-PCV era (1st April 2013 - 31st March 2015), and the numerator is varied using two financial years since the introduction of PCV13. The ratios of case ratios are plotted on a log scale.*

#### Pneumonia of unspecified causative organism


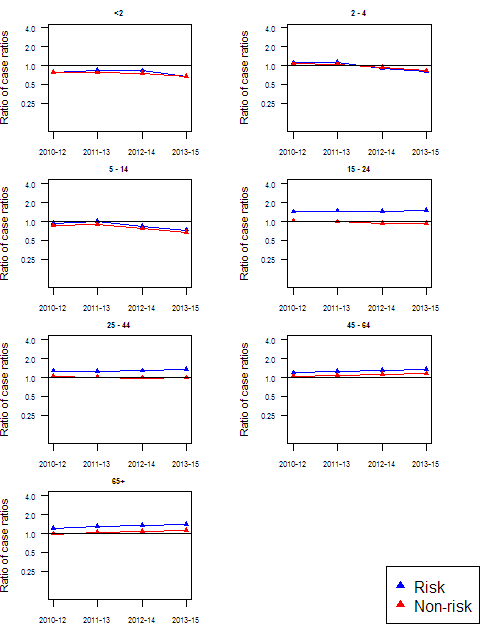


***Figure S11****: Estimated age-specific ratios of case ratios for pneumonia of unspecified causative organism split by risk group. The denominator refers to cases for the pre-PCV era (1st April 2004 - 31st March 2006) and post-PCV era (1st April 2013 - 31st March 2015), and the numerator is varied using two financial years since the introduction of PCV13. The ratios of case ratios are plotted on a log scale.*

## Case ratios for the composite control and risk groups

| Case ratios | Controls for risk groups (min-max) | Controls for non-risk groups (min-max) |
| --- | --- | --- |
| **<2 years** | 1.98 (1.45 - 2.69) | 1.09 (0.84 - 1.43) |
| **2 - 4** | 2.16 (1.63 - 2.71) | 1.03 (0.69 - 1.27) |
| **5 - 14** | 1.78 (1.37 - 2.48) | 1.05 (0.84 - 1.16) |
| **15 - 24** | 1.44 (1.00 - 1.83) | 1.19 (0.85 - 1.60) |
| **25 - 44** | 1.65 (1.46 - 1.94) | 1.17 (0.98 - 1.60) |
| **45 - 64** | 2.14 (1.85 - 2.68) | 1.28 (1.16 - 1.77) |
| **65+** | 2.10 (1.62 - 2.95) | 1.00 (0.86 - 1.14) |

***Table S8****: Age-specific case ratios for the composite control, split by risk group.*

## Additional information outside the direct scope of our analysis

| ICD-10 | Total | <2 yrs | 2 - 4 | 5 - 14 | 15 - 24 | 25 - 44 | 45 - 64 | 65+ |
| --- | --- | --- | --- | --- | --- | --- | --- | --- |
| All-cause pneumonia | 1,771,924 | 52,538 | 47,167 | 37,238 | 27,461 | 113,710 | 257,552 | 1,228,713 |
| J09 | 29  (0%) | 1 (0%) | 0 (0%) | 0 (0%) | 0 (0%) | 7  (0%) | 10 (0%) | 11  (0%) |
| J10 | 4,970 (0%) | 347 (1%) | 190 (0%) | 195 (1%) | 242 (1%) | 997 (1%) | 1,401 (1%) | 1,536 (0%) |
| J11 | 1,656 (0%) | 103 (0%) | 50 (0%) | 51 (0%) | 84 (0%) | 271 (0%) | 308 (0%) | 785 (0%) |
| J12 | 9,529 (1%) | 3245 (6%) | 1,806 (4%) | 731 (2%) | 294 (1%) | 823 (1%) | 1118 (0%) | 1,443 (0%) |
| J13 | 30,459 (2%) | 719 (1%) | 606 (1%) | 610 (2%) | 927 (3%) | 4709 (4%) | 7833 (3%) | 14,523 (1%) |
| J14 | 9095 (1%) | 301 (1%) | 110 (0%) | 141 (0%) | 134 (0%) | 672 (1%) | 2039 (1%) | 5,623 (0%) |
| J15 | 50,263 (3%) | 1,665 (3%) | 1,899 (4%) | 2,308 (6%) | 1,453 (5%) | 5,232 (5%) | 10,080 (4%) | 27,171 (2%) |
| J16 | 1,045 (0%) | 41 (0%) | 19 (0%) | 18 (0%) | 27 (0%) | 90 (0%) | 204 (0%) | 637 (0%) |
| J17 | 876 (0%) | 11 (0%) | 10 (0%) | 8 (0%) | 18 (0%) | 66 (0%) | 232 (0%) | 486 (0%) |
| J18 | 1,683,478 (95%) | 46,823 (89%) | 43,035 (91%) | 33720 (91%) | 24651 (90%) | 102447 (90%) | 238240 (93%) | 1,188,268 (97%) |
|  |  |  |  |  |  |  |  |  |

***Table S9****: Number of admissions for all J09-18 ICD-10 codes over the study period.*
